# Supplementary material for: Sensing of autoinducer-2 by functionally distinct receptors in prokaryotes
Source: Nat Commun. 2020 Oct 23;11:5371. doi: 10.1038/s41467-020-19243-5 (PMC7584622; doi:10.1038/s41467-020-19243-5)
Supplement: Supplementary file 1 — Supplementary Information [file 41467_2020_19243_MOESM1_ESM.pdf]

Supplementary Information

Sensing of autoinducer-2 by functionally distinct receptors in prokaryotes

This PDF file includes:

Supplementary Figures 1-16

Supplementary Tables 1-2

Supplementary References

```
PctA-LBD    --NDYLQRNAIREDLES----YLREMGDVTSSNIQNWLG----------GRLLLVEQTAQ
TlpQ-LBD    QHSSVLVKSASTQMLDESARLRLEARGELQALRIQRYFMDAFQYGKGFSRQILFLRDQAQ

PctA-LBD    T-------LARDHSPETVSALLEQPALTSTFSFTYLGQQDGVFTMRPDSPMPAGYD----
TlpQ-LBD    KRFLDAYDLREDLTRQVRTALAANPEVLGLYVVFEPNALDGKDELFVDQPALGSNDKGRF

PctA-LBD    ----PRSRP---------------------------WYKDAVAAGGLTLTEPYVDAAT--
TlpQ-LBD    SLYWAQATPGQLESESMIESELADTSSGPSGAAYNAWYTCPKESGQPCVLDPYFDKVGER

PctA-LBD    QELIITAATPVKAAGNTLGVVGGDLSLKTLVQIINSLD---FSGMGYAFLVSGDGKILVH
TlpQ-LBD    QLLMTSIAFPLELDGKVIGVMGLDINLSNLQALSEQGNRELYDGVGQVGILSPAGLFAGN

PctA-LBD    P-DKEQVMKTLSEVYPQNTPKIATGFSEAE---LHGHTRILAFTPIKGLP-SVTWYLALS
TlpQ-LBD    SRDAGLLGKNLAKADPQHAGELLQLLAAGKSRLFNENDDLKVLQPLQPIPGAKPWGVLLE

PctA-LBD    IDKDKAY-------AMLSKFRVSA-
TlpQ-LBD    VPKSALLGPALALERQLDDMRREGT
```

Supplementary Fig. 1 Sequence alignment of the LBDs of PctA and TlpQ

Amino acids 30-278 and 36-360 of PctA and TlpQ, respectively, were subjected to multiple sequence alignment using the ClustalW tool of the NPSA suite (https://npsa-prabi.ibcp.fr/cgi-bin/npsa_automat.pl?page=/NPSA/npsa_server.html). Red, identical; green, highly similar; blue, weakly similar. Known key residues involved in interactions with ligands in the binding pockets of PctA-LBD[1,2] and TlpQ-LBD[3] are highlighted in yellow.

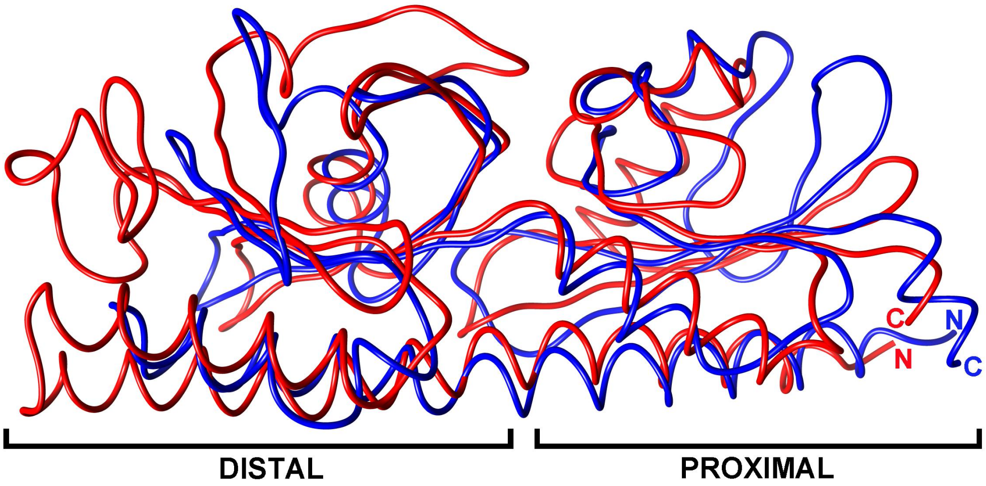

Supplementary Fig. 2 Structural alignment of the LBDs of PctA and TlpQ

The 3D structures of PctA-LBD (PDB ID: 5LTX)[2] and TlpQ-LBD (PDB ID: 6FU4)[3] were aligned using TM-align[4]. The TM-score was normalized by the length of PctA-LBD. Structures of PctA-LBD and TlpQ-LBD are colored in blue and red, respectively. The N- and C-termini as well as the membrane-proximal and membrane-distal modules of PctA-LBD and TlpQ-LBD are labeled.

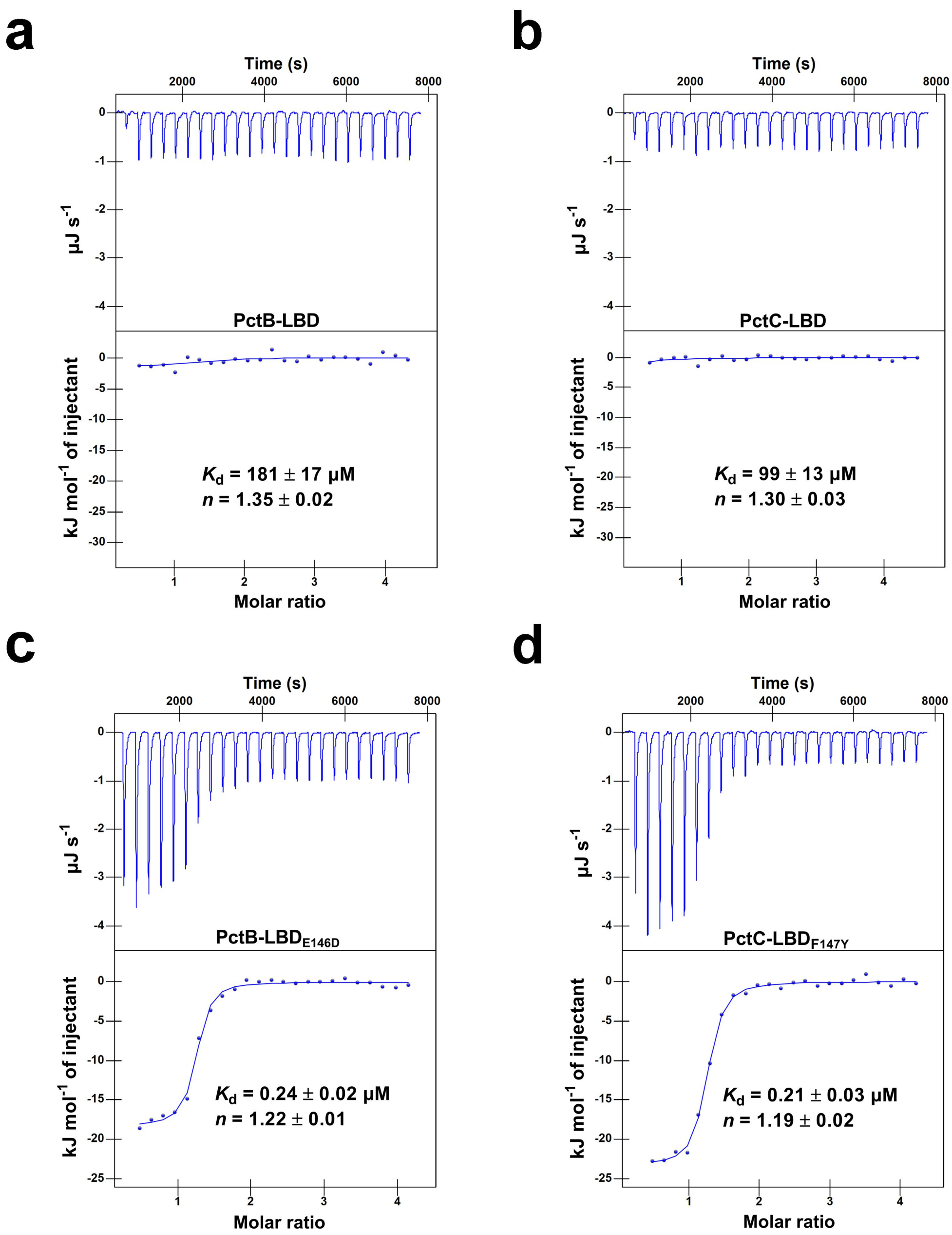

Supplementary Fig. 3 AI-2 shows low affinity to PctB-LBD and PctC-LBD but high affinity to PctB-LBD$_{E146D}$ and PctC-LBD$_{F147Y}$

The binding affinity was evaluated using ITC analysis. ITC data and plots of injected heat for injections of 700 μM DPD/AI-2 into the sample cell containing 70 μM PctB-LBD (**a**), PctC-LBD (**b**), PctB-LBD$_{E146D}$ (**c**) or PctC-LBD$_{F147Y}$ (**d**) are shown in the upper and lower plots, respectively. The heats of ligand dilution were subtracted from the heats of injection and the corrected data were fit to a one-site binding model to determine the K_d values and binding stoichiometry (*n*) by the NanoAnalyze software. Although minor heats were observed for the titration of PctB-LBD (**a**) or PctC-LBD (**b**) with DPD/AI-2, in the context of the suboptimal dilution control (that cannot be changed since ligands are a mixture of interchangeable compounds) it is not certain whether these heats do indeed reflect binding. Data shown are one representative of three independent experiments with similar results, and the K_d and *n* values are presented as mean ± s.d. of the three independent experiments.

```
               10        20        30        40        50        60
                |         |         |         |         |         |
Tsr     MLKRIK-----IVTSLLLVLAVFGLLQLTSGGLFFNALKNDKENFTVLQ---TIRQQQST
PctA    MIKSLKFSHKILLAASLVVFAAFALFTLYNDYLQRNAIREDLESYLREMGDVTSSNIQNW

               70        80        90       100       110       120
                |         |         |         |         |         |
Tsr     LNGSWVALLQTRNTLNRAG----IRYMMDQNNIGSGSTVAELMES-ASISLKQAEKNWAD
PctA    LGGRLLLVEQTAQTLARDHSPETVSALLEQPALTSTFSFTYLGQQDGVFTMRPDSPMPAG

              130       140       150       160       170       180
                |         |         |         |         |         |
Tsr     YEALPRDPRQSTAAAAEIKR------------------------------NYDIYHNALA
PctA    YDPRSRPWYKDAVAAGGLTLTEPYVDAATQELIITAATPVKAAGNTLGVVGGDLSLKTLV

              190       200       210       220       230       240
                |         |         |         |         |         |
Tsr     ELIQLLGAG-------------------------KINEFFDQPTQGYQDGFEKQYVAYME
PctA    QIINSLDFSGMGYAFLVSGDGKILVHPDKEQVMKTLSEVYPQNTPKIATGFSEAELHGHT

              250       260       270       280       290       300
                |         |         |         |         |         |
Tsr     QNDRLHDIAVS-----------DNNASYSQAMWILVGVMIVVLAVIFAVWFG---IKASL
PctA    RILAFTPIKGLPSVTWYLALSIDKDKAYAMLSKFRVSAIAAALISIVAILVLLGLLIRLL

              310       320       330       340       350       360
                |         |         |         |         |         |
Tsr     VAPMNRLIDSIRHIAGG--DLVKPIEVDGSNEMGQLAESLRHMQGELMRTVGDVRNGANA
PctA    MQPLHLMGRAMQDIAQGEGDLTKRLAVTSRDEFGVLGDAFNQFVERIHRSIREVAGTAHK

              370       380       390       400       410       420
                |         |         |         |         |         |
Tsr     IYSGASEIATGNNDLSSRTEQQAASLEETAASMEQLTATVKQNAENARQASHLALSASET
PctA    LHDVSQLVVNASNSSMANSDEQSNRTNSVAAAINELGAAAQEIARNAADASHHASDANHQ

              430       440       450       460       470       480
                |         |         |         |         |         |
Tsr     AQRGGKVVDNVVQTMRDISTSSQK--------------IADIISVIDGIAFQTNILALNA
PctA    AEDGKQVVEQTIRAMNELSEKISASCANIEALNSRTVNIGQILEVIKGISEQTNLLALNA

              490       500       510       520       530       540
                |         |         |         |         |         |
Tsr     AVEAARAGEQGRGFAVVAGEVRNLAQRSAQAAREIKSLIEDS-VGKVDVGSTLVES----
PctA    AIEAARAGEAGRGFAVVADEVRNLAHRAQESAQQIQKMIEELQVGAREAVATMTESQRYS

              550       560       570       580       590       600
                |         |         |         |         |         |
Tsr     ---------AGETMAEIVSAVTRVTDIMGEIASASDEQSRGIDQVGLAVAEMDRVTQQNA
PctA    LESVEIANRAGESLSSVTRRIGEIDGMNQSVATATEEQTAVVDSLNMDITEINTLNQEGV

              610       620       630       640       650       660
                |         |         |         |         |         |
Tsr     ALVEESAAAAAALEEQASRLTEAVAVFRIQQQQRETSAVVKTVTPAAPRKMAVADSEENW
PctA    ENLQATLRACGELETQAGRLRQLVDSFKI-------------------------------

Tsr     ETF
PctA    ---
```

Supplementary Fig. 4 Sequence alignment of PctA from *P. aeruginosa* and the chemoreceptor Tsr from *E. coli*

Amino acid sequence alignment was performed using the ClustalW tool of the NPSA suite (https://npsa-prabi.ibcp.fr/cgi-bin/npsa_automat.pl?page=/NPSA/npsa_server.html). Red, identical; green, highly similar; blue, weakly similar. Five known methylation sites in Tsr[5] are highlighted in yellow and the three potential consensus methylation sites in PctA are highlighted in cyan. The transmembrane regions flanking the ligand binding domains were predicted using the TMHMM Server v. 2.0 (http://www.cbs.dtu.dk/services/TMHMM) and are highlighted in gray.

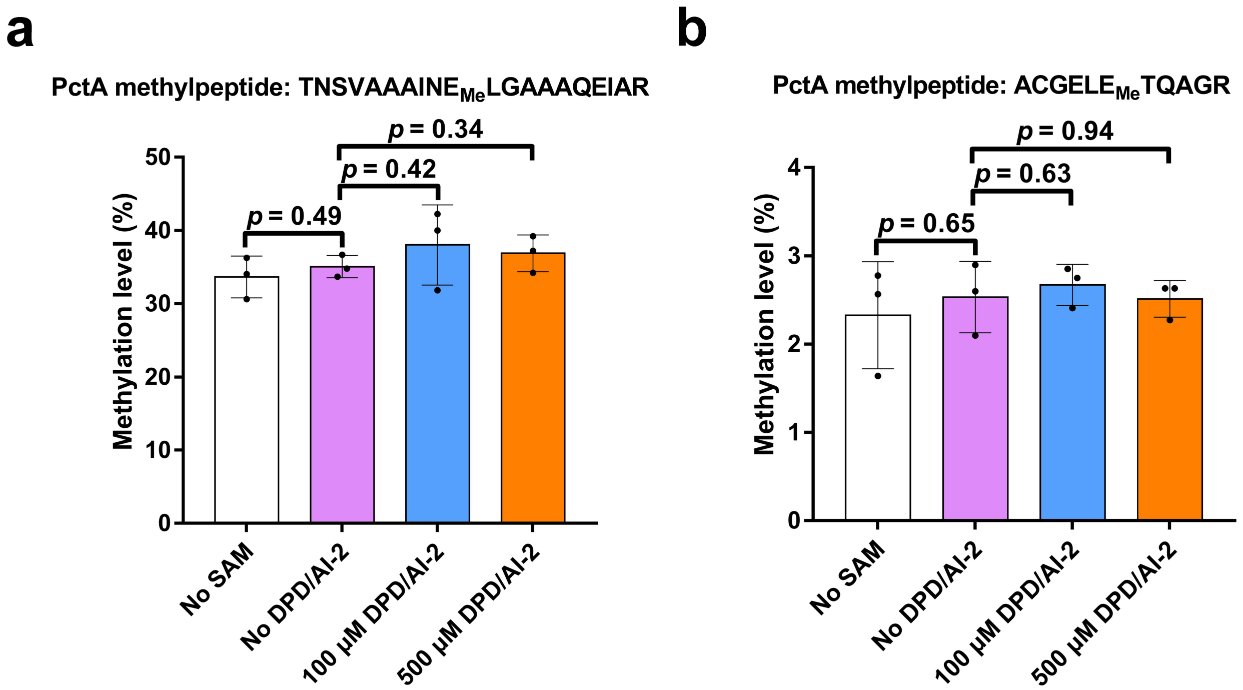

Supplementary Fig. 5 Methylation of PctA at E395 and E614 is not detectably altered by CheR1 in the presence and absence of DPD/AI-2

CheR1-catalyzed methylation of PctA was carried out by co-incubating with SAM in the presence or absence of DPD/AI-2 and reactions without the substrate SAM were established as controls. Quantification of PctA methylation at E395 (**a**) and E614 (**b**) was determined by LC-MS/MS analysis. Data are mean ± s.e.m. of three independent experiments, and statistical significance was determined using two-tailed unpaired Student's *t*-test. *P* values more than 0.05 indicated that the differences between samples are not statistically significant.

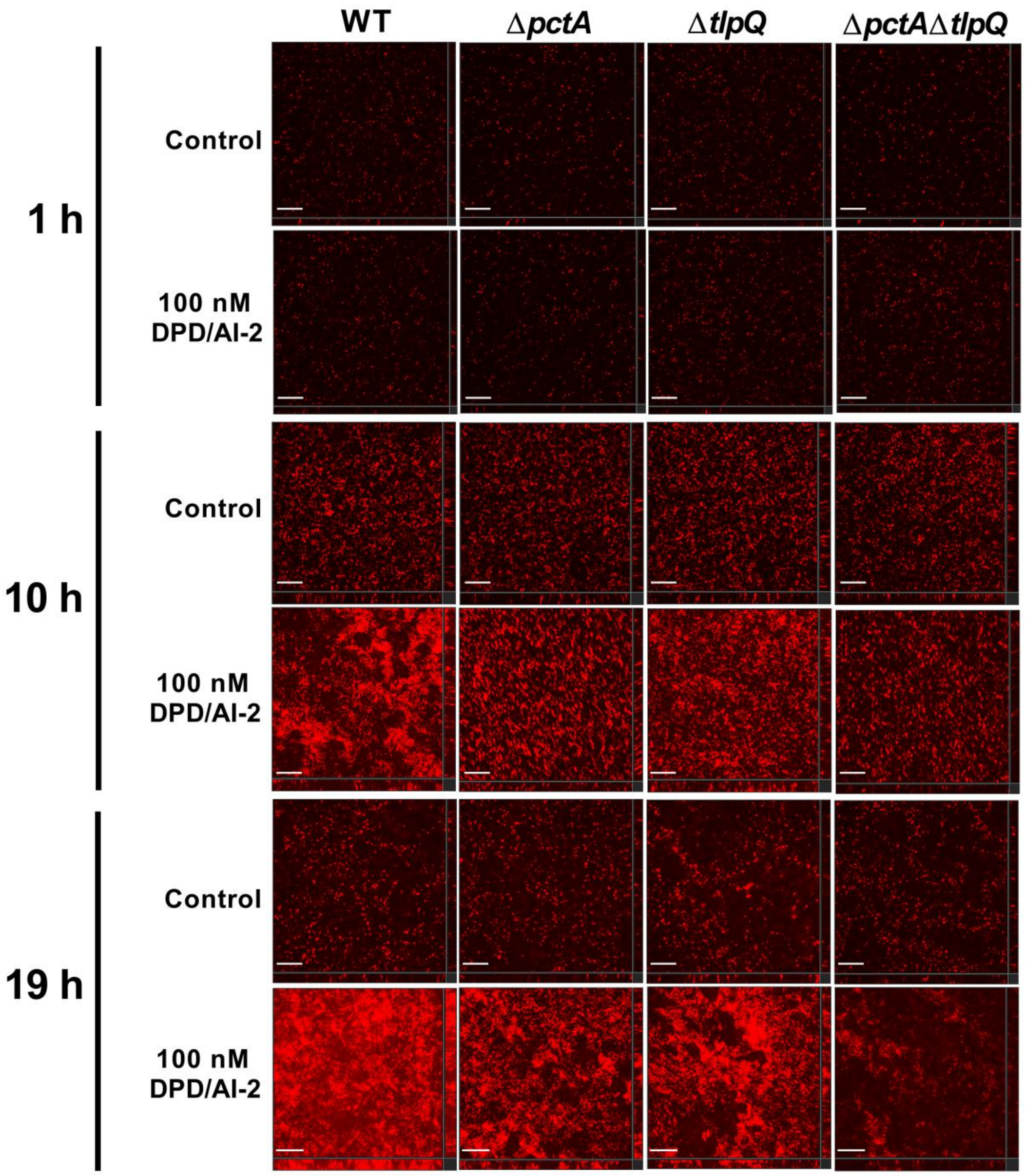

Supplementary Fig. 6 Confocal images of biofilms formed by *P. aeruginosa* strains labeled with mCherry (red)

mCherry-labeled *P. aeruginosa* strains were cultured in TSB medium in the presence or absence of 100 nM DPD/AI-2, and biofilms formed were detected by confocal laser scanning microscopy after incubation at 37°C for 1, 10 and 19 h, respectively. Images are representatives of three independent experiments with similar results. Scale bars, 20 μm. WT, wild-type.

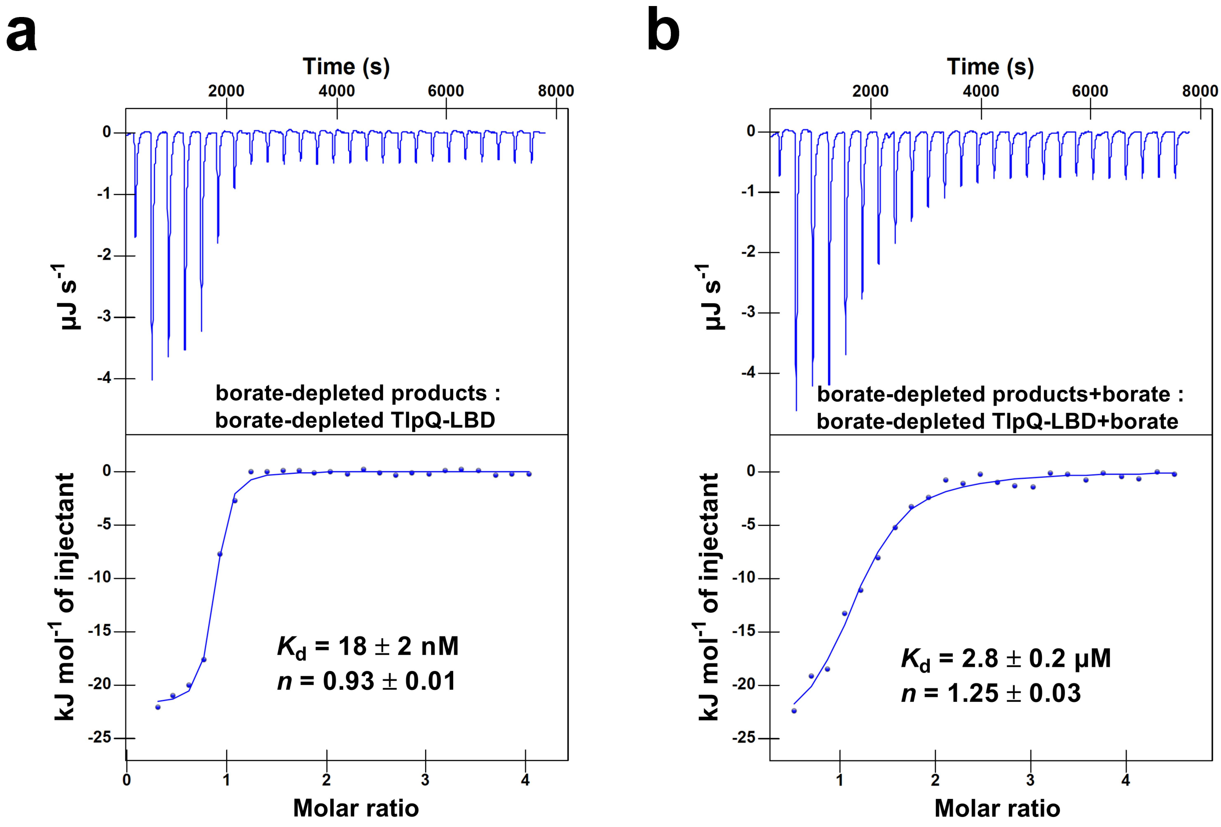

Supplementary Fig. 7 TlpQ-LBD shows higher binding affinity with AI-2 under borate-depleted conditions

The binding affinity was evaluated by ITC analysis. *In vitro* reaction of SAH with Pfs and LuxS was performed with plasticware and borate-depleted water, and DPD/AI-2 concentration in the reaction products is approximately 13 μM. The TlpQ-LBD protein was dialyzed against borate-depleted buffer before use. ITC data and plots of injected heat for automatic injections of the borate-depleted reaction products (**a**) or the borate-depleted products supplemented with 150 μM boric acid (**b**) into the sample cell containing 1.3 μM borate-depleted TlpQ-LBD (**a**) or borate-depleted TlpQ-LBD supplemented with 150 μM boric acid (**b**) are shown in the upper and lower plots, respectively. The binding curves were corrected for the dilution effects in the final analysis. Results shown are one representative of three experiments with similar results. The K_d and binding stoichiometry (*n*) were calculated by the NanoAnalyze software and presented as mean ± s.d. of 3 independent experiments.

a

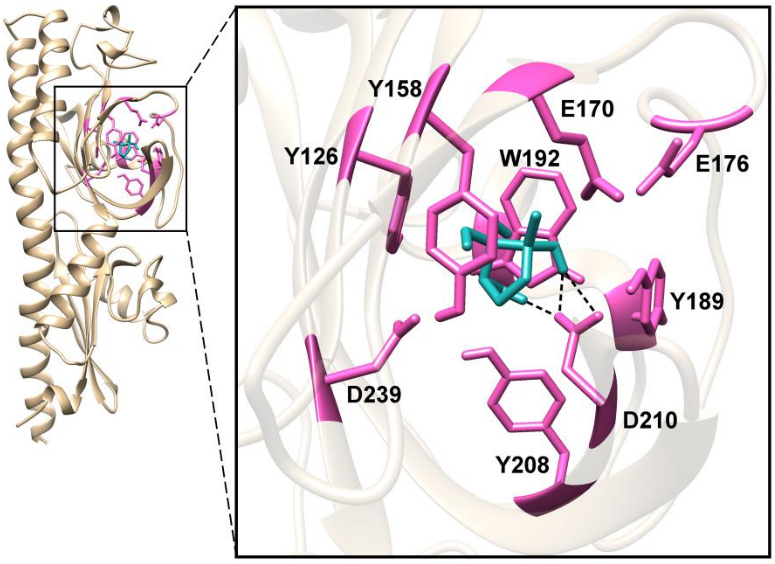

b

Binding affinity

TlpQ-LBD	K_d (μM)
WT	0.12 ± 0.01
W192A	18 ± 3
Y208A	21 ± 2
D210A	45 ± 5
D239A	31 ± 4

Supplementary Fig. 8 Docking analysis of the nonborated *R*-THMF to TlpQ-LBD

a. The predicted conformation of *R*-THMF bound in the histamine-binding pocket of TlpQ-LBD with the lowest docking score. *R*-THMF prepared by LigPrep was docked into TlpQ-LBD (PDB ID: 6FU4)[3] using the Glide XP Docking mode. The best conformation with the lowest docking score is given by using the Chimera software. Residues of TlpQ-LBD in close proximity to *R*-THMF are shown as purple sticks and *R*-THMF is shown as cyan sticks. The three potential hydrogen bonds are indicated by dashed lines.

b. Binding of 700 μM DPD/AI-2 to 70 μM TlpQ-LBD and its mutants. The binding affinity was analyzed using ITC. Data shown are mean ± s.d. of three biological replicates. WT, wild-type.

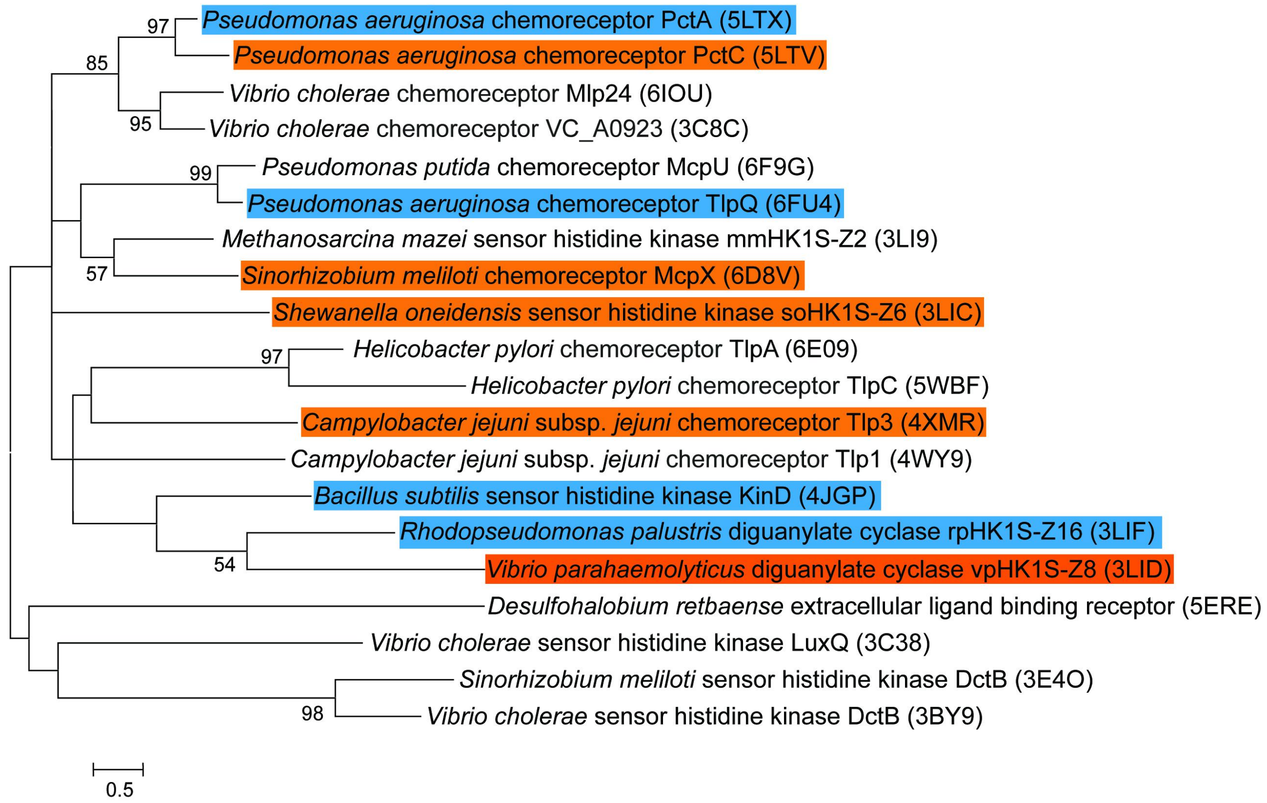

Supplementary Fig. 9 Maximum-likelihood phylogenetic tree showing the relationship of PctA-LBD and 19 bacterial dCACHE domains highly similar in their structures

Multiple sequence alignments were performed with ClustalW and phylogenetic tree was reconstructed by using the maximum-likelihood method based on the Jones-Taylor-Thornton (JTT) model embedded in the MEGA7 software. The PDB IDs of the dCACHE domains are given in parentheses. The dCACHE domains with a high AI-2 binding activity are depicted in blue and those with a low AI-2 binding activity are in red. Bootstrap values (expressed as percentages of 1000 replications) greater than 50% are shown on the branch. Scale bar indicates evolutionary distance of 0.5 amino acid substitutions per position.

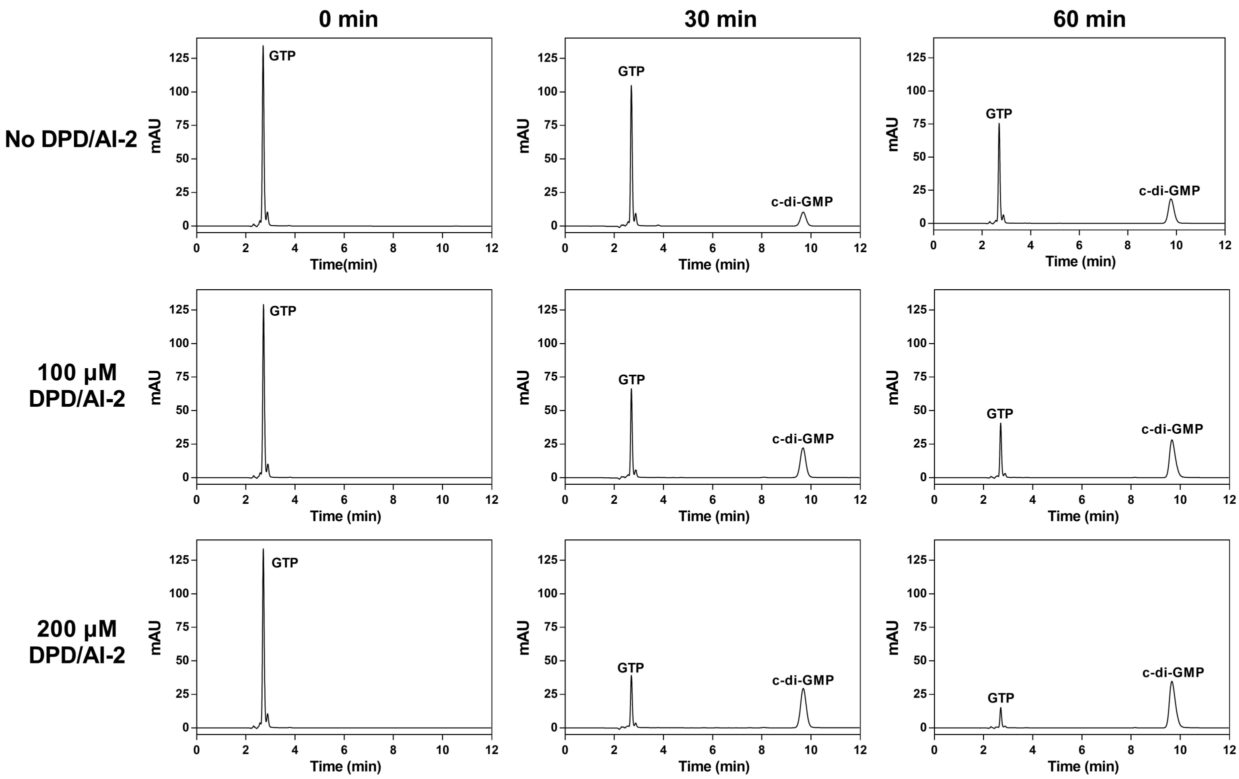

Supplementary Fig. 10 AI-2 induces the DGC activity of rpHK1S-Z16 in c-di-GMP synthesis

Membrane fractions containing rpHK1S-Z16 were incubated with GTP in the presence or absence of DPD/AI-2 at 30°C for 0, 30 and 60 min and the products were analyzed by HPLC. HPLC spectra shown are representatives of three independent experiments with similar results.

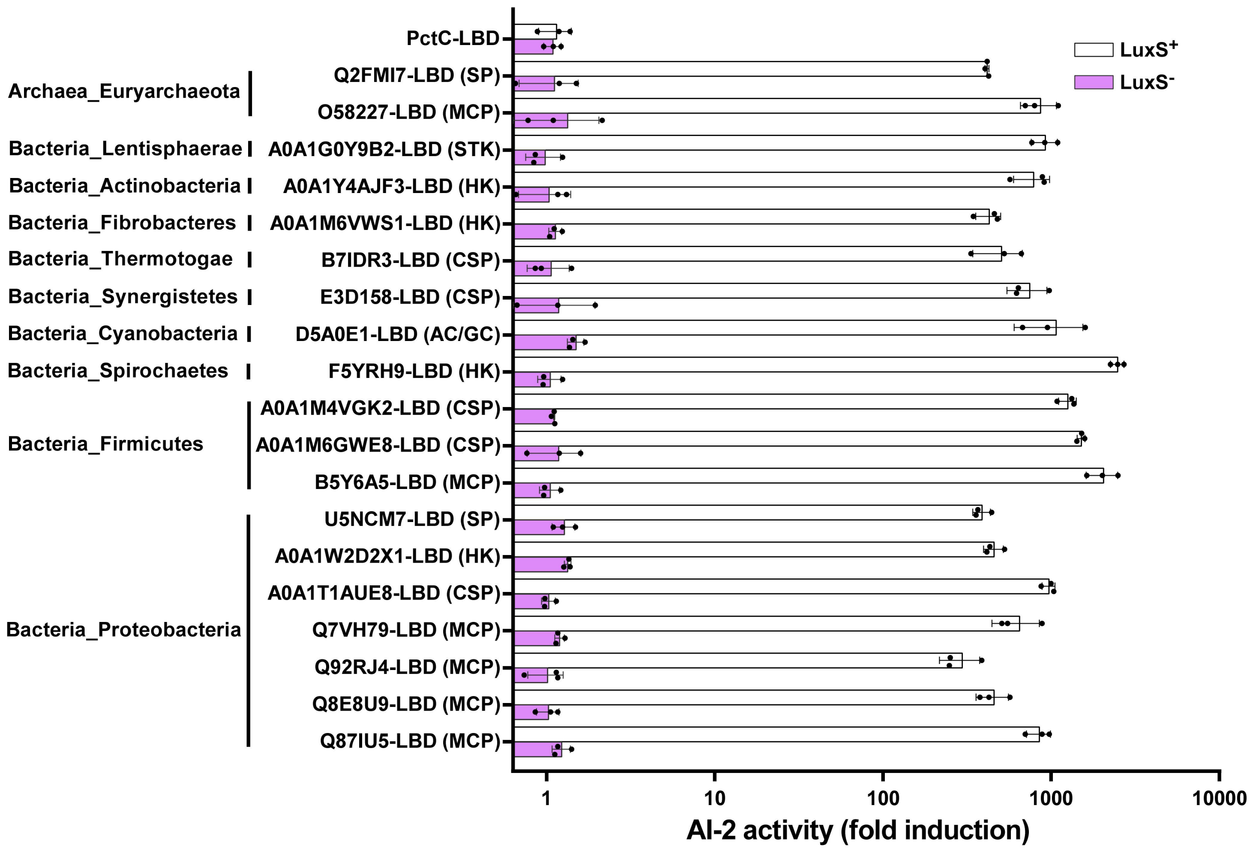

Supplementary Fig. 11 dCache_1 domains from proteins predicted to function as MCPs, HKs, CSPs, SPs, STKs or ACs/GCs in diverse bacteria and archaea are capable of retaining AI-2

Light production by *V. harveyi* strain MM32 was measured following the addition of a buffer control or ligands released from purified dCache_1 domains expressed in a *luxS*$^+$ (white bars) or a *luxS*$^-$ (purple bars) *E. coli* strain. The PctC-LBD showing low binding affinity for AI-2 was used as a negative control. Results are shown as fold induction relative to the light production induced by the buffer control (mean ± s.e.m.; *n*=3 independent experiments). The Uniprot IDs of the 19 dCache_1-containing proteins are provided, and the signal transduction protein families to which they belong are given in parentheses. Phyletic distribution of the 19 dCache_1-containing proteins at the phylum level in prokaryotes is shown.

```
PctA (36-113)          NAIREDLESYLRE........MGDVTSSNIQNWLG.GRLLLVEQTAQTLARDHSPE............TVSALLEQPALTSTFS...FTYLG.QQDGVFTMRP....
PctB (36-113)          ASIREDLEDYLHE........MGEITASNVQNWLS.GRILLIENLAQTLARDHSPE............TTQALLEQPLLGSTFL...FTYLG.QTDGTYTARP....
PctC (36-116)          EAVRTDTENYLGE........IGTLTASNIQSWLE.GRMHLVEGLASQLALLDQPDE..........ANIARQLEQPVFSRNFA...SVYLGEAASGTFTMRP....
TlpQ (50-142)          ......LDESARLRLEARGELQALRIQRYFMDAFQYGKGFSRQILFLRDQ.AQKRFLDAYDLREDLT......RQVRTALAANPEVLGLYVVFEP.NALDGKDELFV
KinD (41-86)           ........DTIAAEHKQEASVLLNLHRNKINYLIGETMARMTSLSIAID....RPVDI................................................
rpHK1S-Z16 (46-89)     ........KIALAQSETEMRNLSHSLAEHATHTFQGADVV.............................................LDDIVSFMKWRP........

PctA (114-149)         ......................................DSPMPAGYDPRSRPW...................YKDAVAAGG.......LTLTE.....PYVDAAT
PctB (114-149)         ......................................TSDLPADYDPRRRPW...................YNAATSAGQ.......TTLTE.....PYMEPAI
PctC (117-152)         ......................................YDAMPEGYDPRTRAW...................YKDALAADR.......LIVTE.....PFVDAGT
TlpQ (143-213)         DQPA....LGSNDKGRFSLYWAQATPGQLESESMIESELADTSSGPSGAAYNAW...................YTCPKESG.......QPCVLD.....PYFDKVG
KinD (87-153)          ..............KKMQSILEKTFDSEP.........RFSGLYFLNAKGDVTASTT.......ELKTKVNLADRSFFIKAKETK.........KTVISDSYSSRI
rpHK1S-Z16 (90-160)    ....HPSP......VFNERLRALADNLPQ...........LSDVAILDADGQLTYASVKPVPALD.....N..SDRSYFRYHRAN.DDH......TLLITGPIQSR.

PctA (150-216)         .......QELIITAATPVKAA..G...NTLGVVGGDLSLKTLVQIIN..SLDFSGMG.............YAFLVSGDGK............ILVHP.DKEQVMKTL
PctB (150-216)         .......HELVLTIASPARQG..G...QPFGVVGGDLSLQTVVKIIN..SLDFGGMG.............YAFLVSGDGK............ILVHP.DKDQVMKSL
PctC (153-219)         .......GEQILAMSLPVRH.....AGQLLGVAAGDMKLETLTAILN..SLKFDGAG.............YAFLVSDAGK............ILLHP.DSGLVLKTL
TlpQ (214-286)         E.RQ....LLMTSIAFPLELDGK.....VIGVMGLDINLSNLQALSEQGNRE............LYDGVGQVGILSPA............GLFAGNSRDAGLLGKNL
KinD (154-238)         TG......QPIFTICVPVLD....SKRNVTDYLVAAIQIDYLKNL............INLLSPDVYIEVVNQDGKMIFASGQASHAEDQKPVSGYLDDISWNMKVYP
rpHK1S-Z16 (161-225)   ....TSGV.WVFVVSRRLETTDG....KFFGVVVATIESEYFS.......TFYKTFD.......LGPGGSISLLH.SDGR...........LLIQWPSLQ.......

PctA (217-261)         SEVY................................PQNTPKIATGFSEAE...LHGHTRILAFTPIKGLPSV.........T.WYLALS
PctB (217-261)         SDVY................................PRNTPKIGSGFSEAE...LHGNTRILSFSPVKGLSGL.........D.WYIGIS
PctC (220-264)         AEAY................................PKGAPNIVPGVHEVE...LDGSSQFVSFTPVKGLPGV.........T.WYVALV
TlpQ (287-335)         AKADPQHA...............................GELLQLLAAG..KSRLFNENDDLKVLQPLQPIP.......GAK.PWGVLLE
KinD (239-252)         NPVTIEELSKSLVL.............................................................................
rpHK1S-Z16 (226-275)   ..............TGRDMANMVLFQKALPRSPDGYYLTV....................SPFDGLTK......YLAYRRVSRYPLVVTV.
```

Supplementary Fig. 12 Multiple sequence alignment of the dCache_1-type LBDs of selected proteins

A total of 18970 dCache_1 domains downloaded from the Pfam 32.0 database were aligned using ClustalW embedded in MEGA7 software (Supplementary Data 3) and the alignment results of the LBDs of PctA, PctB, PctC, TlpQ, KinD and rpHK1S-Z16 were extracted and presented. The conserved residues corresponding to R126, W128, Y144, D146 and D173 of PctA are highlighted in yellow and non-conserved residues in the five positions are highlighted in purple.

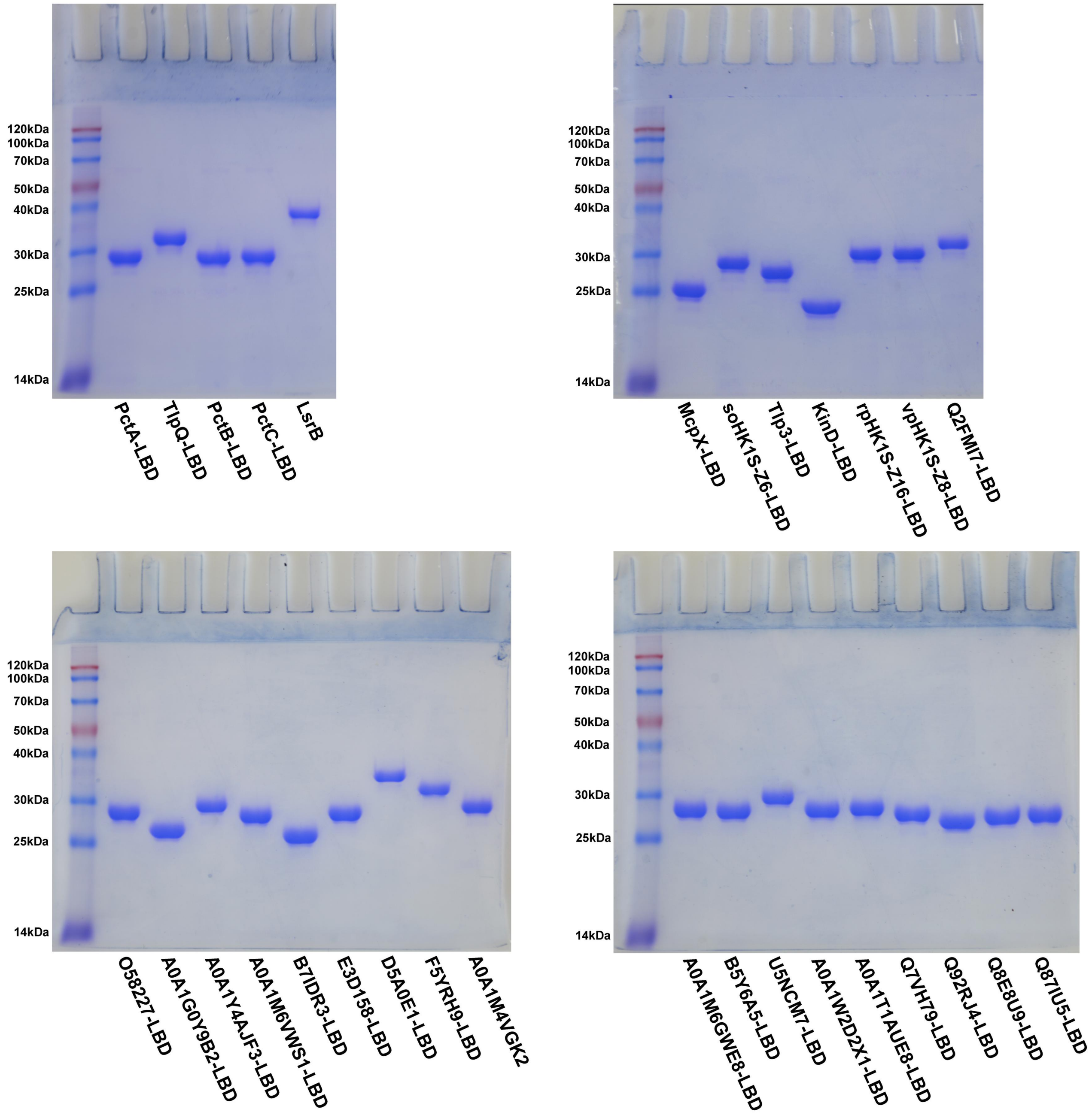

Supplementary Fig. 13 SDS-PAGE gels of purified LBDs and LsrB used for *in vitro* AI-2 binding assays

LsrB and dCACHE-type LBDs with an N-terminal His_6 tag purified by Ni^{2+}-NTA affinity chromatography were subjected to SDS-PAGE analysis before being used in *in vitro* AI-2 binding assays. Representatives of three independent experiments with similar results are shown.

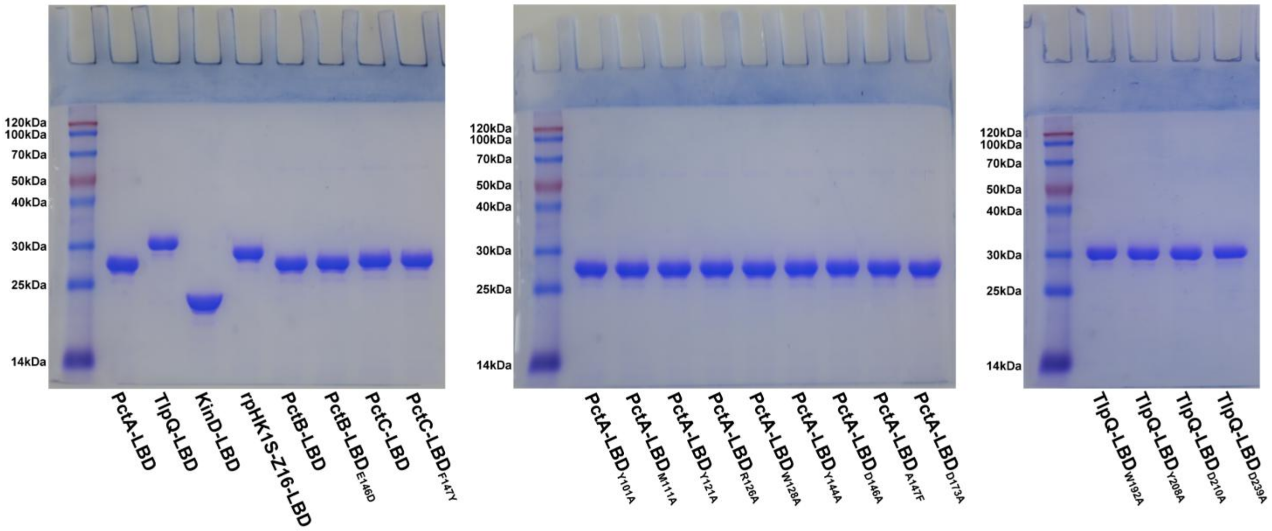

Supplementary Fig. 14 SDS-PAGE gels of purified tag-free LBDs used for ITC experiments

The N-terminal His_6 tags of dCache_1-type LBDs were cleaved off and the tag-free proteins were subjected to SDS-PAGE analysis before being used in ITC experiments. Images shown are representatives of three independent experiments with similar results.

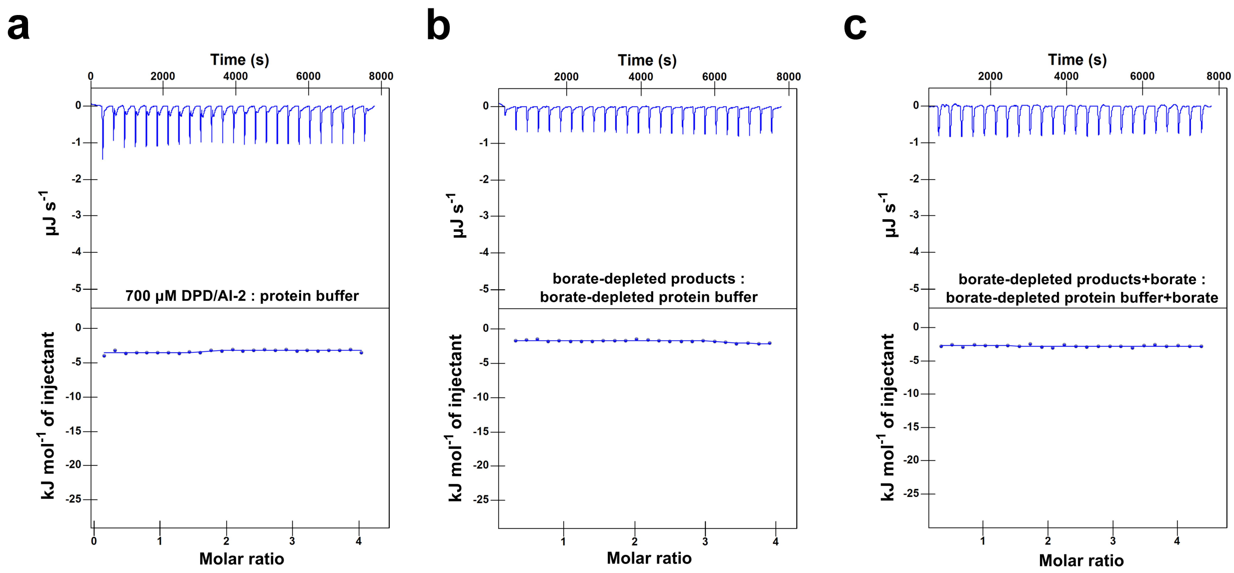

Supplementary Fig. 15 ITC control experiments determining heats of dilution of ligands

In control experiments, protein buffer (**a**), borate-depleted protein buffer (**b**), or borate-depleted protein buffer supplemented with 150 μM boric acid (**c**) in the sample cell was titrated with 700 μM DPD/AI-2 (**a**), the products of the Pfs/LuxS reaction in a borate-depleted system (**b**), or the products of the Pfs/LuxS reaction in a borate-depleted system supplemented with 150 μM boric acid (**c**) to obtain the heat of dilution. Original titration data and integrated normalized values are shown in the upper and lower plots, respectively. Data shown are one representative of three independent experiments with similar results.

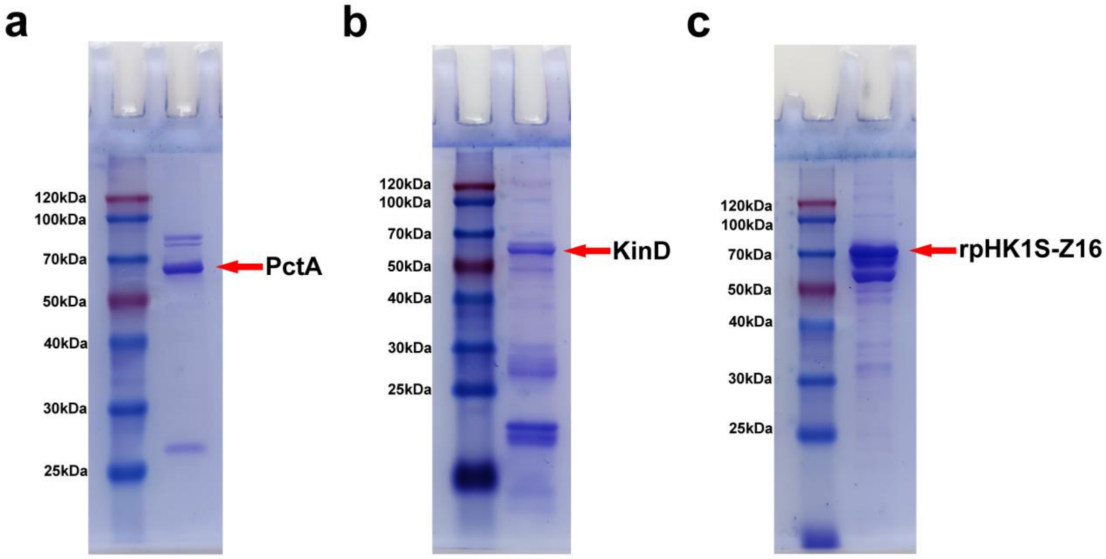

Supplementary Fig. 16 SDS-PAGE gels of purified full-length PctA, KinD and rpHK1S-Z16

After inverted membrane extraction and further purification by Ni^{2+}-NTA affinity chromatography, membrane fractions containing full-length PctA (**a**), KinD (**b**) or rpHK1S-Z16 (**c**) were subjected to SDS-PAGE analysis to examine the purity. Similar results were obtained in three independent experiments.

Supplementary Table 1. Bacterial strains and plasmids used in this study.

Strains and plasmids	Relevant characteristics*	Source
**Strains**		
P. aeruginosa		
PAO1	Wild-type	Laboratory stock
Δ*mcpB*	*mcpB* deletion mutant in PAO1	This study
Δ*cttP*	*cttP* deletion mutant in PAO1	This study
Δ*wspA*	*wspA* deletion mutant in PAO1	This study
Δ*pilJ*	*pilJ* deletion mutant in PAO1	This study
Δ*PA1251*	*PA1251* deletion mutant in PAO1	This study
Δ*bdlA*	*bdlA* deletion mutant in PAO1	This study
Δ*aer*	*aer* deletion mutant in PAO1	This study
Δ*PA1608*	*PA1608* deletion mutant in PAO1	This study
Δ*PA1646*	*PA1646* deletion mutant in PAO1	This study
Δ*mcpS*	*mcpS* deletion mutant in PAO1	This study
Δ*ctpH*	*ctpH* deletion mutant in PAO1	This study
Δ*PA2573*	*PA2573* deletion mutant in PAO1	This study
Δ*PA2562*	*PA2562* deletion mutant in PAO1	This study
Δ*tlpQ*	*tlpQ* deletion mutant in PAO1	This study
Δ*PA2788*	*PA2788* deletion mutant in PAO1	This study
Δ*PA2867*	*PA2867* deletion mutant in PAO1	This study
Δ*PA2920*	*PA2920* deletion mutant in PAO1	This study
Δ*PA4290*	*PA4290* deletion mutant in PAO1	This study
Δ*pctC*	*pctC* deletion mutant in PAO1	This study
Δ*pctA*	*pctA* deletion mutant in PAO1	This study
Δ*pctB*	*pctB* deletion mutant in PAO1	This study
Δ*PA4520*	*PA4520* deletion mutant in PAO1	This study
Δ*PA4633*	*PA4633* deletion mutant in PAO1	This study
Δ*ctpL*	*ctpL* deletion mutant in PAO1	This study
Δ*PA4915*	*PA4915* deletion mutant in PAO1	This study
Δ*mcpK*	*mcpK* deletion mutant in PAO1	This study
Δ*pctA*Δ*tlpQ*	*pctA*/*tlpQ* double deletion mutant in PAO1	This study
B. subtilis		
B. subtilis 168	Wild-type	Laboratory stock
R. palustris		
R. palustris R1	Wild-type	China General Microbiological Culture Collection Center
S. meliloti		
S. meliloti CCNWSX0020	Wild-type	6

S. oneidensis		
S. oneidensis MR-1	Wild-type	7
V. parahaemolyticus		
V. parahaemolyticus ATCC 17802	Wild-type	8
E. coli		
BL21(DE3)	Host for expression vector pET-28a	Novagen
$LuxS^-$ BL21(DE3)	Δ*luxS* deletion mutant in *E. coli* BL21(DE3)	This study
XL1-Blue	Host for expression vector pGEX-6P-1	Novagen
TG1	Host for cloning	Stratagene
S17-1	F^- *thi pro hsdR* [RP4-2 Tc::Mu Km::Tn7 (Tp Sm)]	9
HCB721	Host for expression of *pctA* of *P .aeruginosa*	10
V. harveyi		
V. harveyi MM32	*luxN*::*cat luxS*::Tn*5kan*	11
**Plasmids**		
p34S-Gm	Gm resistant cassette carrying vector; Ap^r, Gm^r	12
pK18*mobsacB*	*sacB*-based gene replacement vector; Km^r	13
pK18-Δ*mcpB*	Δ*mcpB*::Gm in pK18*mobsacB* ; Km^r, Gm^r	This study
pK18-Δ*cttP*	Δ*cttP*::Gm in pK18*mobsacB* ; Km^r, Gm^r	This study
pK18-Δ*wspA*	Δ*wspA*::Gm in pK18*mobsacB* ; Km^r, Gm^r	This study
pK18-Δ*pilJ*	Δ*pilJ*::Gm in pK18*mobsacB* ; Km^r, Gm^r	This study
pK18-Δ*PA1251*	Δ*PA1251*::Gm in pK18*mobsacB* ; Km^r, Gm^r	This study
pK18-Δ*bdlA*	Δ*bdlA*::Gm in pK18*mobsacB* ; Km^r, Gm^r	This study
pK18-Δ*aer*	Δ*aer*::Gm in pK18*mobsacB* ; Km^r, Gm^r	This study
pK18-Δ*PA1608*	Δ*PA1608*::Gm in pK18*mobsacB* ; Km^r, Gm^r	This study
pK18-Δ*PA1646*	Δ*PA1646*::Gm in pK18*mobsacB* ; Km^r, Gm^r	This study
pK18-Δ*mcpS*	Δ*mcpS*::Gm in pK18*mobsacB* ; Km^r, Gm^r	This study
pK18-Δ*ctpH*	Δ*ctpH*::Gm in pK18*mobsacB* ; Km^r, Gm^r	This study
pK18-Δ*PA2573*	Δ*PA2573*::Gm in pK18*mobsacB* ; Km^r, Gm^r	This study
pK18-Δ*PA2562*	Δ*PA2562*::Gm in pK18*mobsacB* ; Km^r, Gm^r	This study
pK18-Δ*tlpQ*	Δ*tlpQ*::Gm in pK18*mobsacB* ; Km^r, Gm^r	This study
pK18-Δ*PA2788*	Δ*PA2788*::Gm in pK18*mobsacB* ; Km^r, Gm^r	This study
pK18-Δ*PA2867*	Δ*PA2867*::Gm in pK18*mobsacB* ; Km^r, Gm^r	This study
pK18-Δ*PA2920*	Δ*PA2920*::Gm in pK18*mobsacB* ; Km^r, Gm^r	This study
pK18-Δ*PA4290*	Δ*PA4290*::Gm in pK18*mobsacB* ; Km^r, Gm^r	This study
pK18-Δ*pctC*	Δ*pctC*::Gm in pK18*mobsacB* ; Km^r, Gm^r	This study
pK18-Δ*pctA*	Δ*pctA*::Gm in pK18*mobsacB* ; Km^r, Gm^r	This study
pK18-Δ*pctB*	Δ*pctB*::Gm in pK18*mobsacB* ; Km^r, Gm^r	This study
pK18-Δ*PA4520*	Δ*PA4520*::Gm in pK18*mobsacB* ; Km^r, Gm^r	This study
pK18-Δ*PA4633*	Δ*PA4633*::Gm in pK18*mobsacB* ; Km^r, Gm^r	This study
pK18-Δ*ctpL*	Δ*ctpL*::Gm in pK18*mobsacB* ; Km^r, Gm^r	This study

pK18-Δ*PA4915*	Δ*PA4915*::Gm in pK18*mobsacB* ; Km^r, Gm^r	This study
pK18-Δ*mcpK*	Δ*mcpK*::Gm in pK18*mobsacB* ; Km^r, Gm^r	This study
pME6032	Shuttle vector containing $lacI^q$-*Ptac* fragment for gene expression; source of *tetA* gene cassette, Tc^r	14
pME6032-*pctA*	*pctA* cloned into pME6032 for complementation	This study
pME6032-*tlpQ*	*tlpQ* cloned into pME6032 for complementation	This study
pME6032-*pctB*	pME6032 expressing *pctB*	This study
pME6032-mCherry	pME6032 expressing mCherry	This study
pET-28a	Expression vector with N-terminal hexahistidine affinity tag, Km^r	Novagen
pET28a-*pctA-LBD*	pET-28a expressing *pctA-LBD*	This study
pET28a-*pctB-LBD*	pET-28a expressing *pctB-LBD*	This study
pET28a-*pctC-LBD*	pET-28a expressing *pctC-LBD*	This study
pET28a-*tlpQ-LBD*	pET-28a expressing *tlpQ-LBD*	This study
pET28a-*mcpX-LBD*	pET-28a expressing *mcpX-LBD*	This study
pET28a-*soHK1S-Z6-LBD*	pET-28a expressing *soHK1S-Z6-LBD*	This study
pET28a-*vpHK1S-Z8-LBD*	pET-28a expressing *vpHK1S-Z8-LBD*	This study
pET28a-*kinD-LBD*	pET-28a expressing *kinD-LBD*	This study
pET28a-*rpHK1S-Z16-LBD*	pET-28a expressing *rpHK1S-Z16-LBD*	This study
pET28a-*tlp3-LBD*	pET-28a expressing *tlp3-LBD*	This study
pET28a-*lsrB*	pET-28a expressing *lsrB*	This study
pET28a-*pctA-LBD*^{Y101A}	pET-28a expressing *pctA-LBD*^{Y101A}	This study
pET28a-*pctA-LBD*^{M111A}	pET-28a expressing *pctA-LBD*^{M111A}	This study
pET28a-*pctA-LBD*^{Y121A}	pET-28a expressing *pctA-LBD*^{Y121A}	This study
pET28a-*pctA-LBD*^{R126A}	pET-28a expressing *pctA-LBD*^{R126A}	This study
pET28a-*pctA-LBD*^{W128A}	pET-28a expressing *pctA-LBD*^{W128A}	This study
pET28a-*pctA-LBD*^{Y144A}	pET-28a expressing *pctA-LBD*^{Y144A}	This study
pET28a-*pctA-LBD*^{D146A}	pET-28a expressing *pctA-LBD*^{D146A}	This study
pET28a-*pctA-LBD*^{A147F}	pET-28a expressing *pctA-L*BD^{A147F}	This study
pET28a-*pctA-LBD*^{D173A}	pET-28a expressing *pctA-LBD*^{D173A}	This study
pET28a-*pctB-LBD*^{E146D}	pET-28a expressing *pctB-LBDE*^{146D}	This study
pET28a-*pctC-LBD*^{F147Y}	pET-28a expressing *pctC-LBD*^{F147Y}	This study
pET28a-*tlpQ-LBD*^{W192A}	pET-28a expressing *tlpQ-LBD*^{W192A}	This study
pET28a-*tlpQ-LBD*^{Y208A}	pET-28a expressing *tlpQ-LBD*^{Y208A}	This study
pET28a-*tlpQ-LBD*^{D210A}	pET-28a expressing *tlpQ-LBD*^{D210A}	This study
pET28a-*tlpQ-LBD*^{D239A}	pET-28a expressing *tlpQ-LBD*^{D239A}	This study
pET28a-*cheR1*	pET-28a expressing *cheR1*	This study
pET28a-*Q87IU5-LBD*	pET-28a expressing *Q87IU5-LBD*	This study
pET28a-*Q8E8U9-LBD*	pET-28a expressing *Q8E8U9-LBD*	This study
pET28a-*Q92RJ4-LBD*	pET-28a expressing *Q92RJ4-LBD*	This study
pET28a-*U5NCM7-LBD*	pET-28a expressing *U5NCM7-LBD*	This study
pET28a-*A0A1T1AUE8-LBD*	pET-28a expressing *A0A1T1AUE8-LBD*	This study

pET28a-*A0A1W2D2X1-LBD*	pET-28a expressing *A0A1W2D2X1-LBD*	This study
pET28a-*Q7VH79-LBD*	pET-28a expressing *Q7VH79*-LBD	This study
pET28a-*A0A1M4VGK2-LBD*	pET-28a expressing *A0A1M4VGK2-LBD*	This study
pET28a-*O58227-LBD*	pET-28a expressing *O58227-LBD*	This study
pET28a-Q2FMI7-LBD	pET-28a expressing *Q2FMI7-LBD*	This study
pET28a-*A0A1M6VWS1*-LBD	pET-28a expressing *A0A1M6VWS1-LBD*	This study
pET28a-*B5Y6A5-LBD*	pET-28a expressing *B5Y6A5-LBD*	This study
pET28a-*B7IDR3-LBD*	pET-28a expressing *B7IDR3-LBD*	This study
pET28a-*E3D158-LBD*	pET-28a expressing *E3D158-LBD*	This study
pET28a-*F5YRH9-LBD*	pET-28a expressing *F5YRH9-LBD*	This study
pET28a-*A0A1Y4AJF3-LBD*	pET-28a expressing *A0A1Y4AJF3-LBD*	This study
pET28a-*D5A0E1-LBD*	pET-28a expressing *D5A0E1*-LBD	This study
pET28a-*A0A1M6GWE8-LBD*	pET-28a expressing *A0A1M6GWE8-LBD*	This study
pET28a-*A0A1G0Y9B2-LBD*	pET-28a expressing *A0A1G0Y9B2-LBD*	This study
pGEX-6P-1	Expression vector with N-terminal GST tag, Amp^r	Novagen
pGEX-6P-1-*pfs*	pGEX-6P-1 expressing *pfs*	This study
pGEX-6P-1-*luxS*	pGEX-6P-1 expressing *luxS*	This study
pHSe5	the *E. coli* expression plasmid pHSe5, Ap^r	10
pHSe5-*pctA-his*$_6$	pHSe5 expressing *pctA* with a C-terminal His_6 tag	This study
pHSe5-*kinD-his*$_6$	pHSe5 expressing *kinD* with a C-terminal His_6 tag	This study
pHSe5-*rpHK1S-Z16-his*$_6$	pHSe5 expressing *rpHK1S-Z16* with a C-terminal His_6 tag	This study
pCas	Crispr-Cas9 system plasmid used for in-frame deletion, Km^r	15
pTargetF	CRISPR-Cas9 system plasmid used for in-frame deletion, Sp^r	15
pTargetF1	pTargetF with the spectinomycin resistance gene replaced by a chloramphenicol resistance gene, Cm^r	16
pTargetF1-Δ*luxS*	pTargetF1 derivative for *luxS* deletion in *E. coli* BL21(DE3)	This study

***Tc^r, Gm^r, Km^r, Cm^r, Sp^r and Ap^r represent resistance to tetracycline, gentamicin, kanamycin, chloramphenicol, spectinomycin and ampicillin, respectively.**

Supplementary Table 2. Primers used in this study.

Primers	5'-3' sequence*	
Gm-F	CCCAAGCTTTTGACATAAGCCTGTTCGG	To generate the gentamicin resistance cassette
Gm-R	CCCAAGCTTTTAGGTGGCGGTACTTGG	
Δ*mcpB*-up-F	GGAAACAGCTATGACCATGATTACGAATTCAATTTCCGCCGCGTGGAG	To generate pK18-Δ*mcpB*
Δ*mcpB*-up-R	GCCCGCAGGCTGTCGTAGA	
Δ*mcpB*-down-F	TCTACGACAGCCTGCGGGCGGTGTTCAGGCTGGACACTCCG	
Δ*mcpB*-down-R	GCATGCCTGCAGGTCGACTCTAGAGGATCCCACCAGTTGCCGCAGTTCCTC	
Δ*cttP*-up-F	GGAAACAGCTATGACCATGATTACGAATTCCCCACCTACCTCCGCGACAA	To generate pK18-Δ*cttP*
Δ*cttP*-up-R	TGCGGGTTGGCATTCGCTA	
Δ*cttP*-down-F	TAGCGAATGCCAACCCGCAGGAATGGCCCGAACGGGT	
Δ*cttP*-down-R	GCATGCCTGCAGGTCGACTCTAGAGGATCCTCATATCCTGGGGCATGGGAC	
Δ*wspA*-up-F	GGAAACAGCTATGACCATGATTACGAATTCATCGCCATCATCTTCGCTCTG	To generate pK18-Δ*wspA*
Δ*wspA*-up-R	AGTTGTCGGTCCACGAACTGC	
Δ*wspA*-down-F	GCAGTTCGTGGACCGACAACTAGATGGTCAACGAGGGTATGCAG	
Δ*wspA*-down-R	GCATGCCTGCAGGTCGACTCTAGAGGATCCTCGTTCATGCCGTCCCCTC	
Δ*pilJ*-up-F	GGAAACAGCTATGACCATGATTACGAATTCATGGGCGGGCGGTTCTTC	To generate pK18-Δ*pilJ*
Δ*pilJ*-up-R	CAGGATGTTCCAGCGTTTCTCG	
Δ*pilJ*-down-F	CGAGAAACGCTGGAACATCCTGCCATCCAGACCGACACCAACG	
Δ*pilJ*-down-R	GCATGCCTGCAGGTCGACTCTAGAGGATCCCACATCGCCAGCGAGTAGGGTT	
Δ*PA1251*-up-F	GGAAACAGCTATGACCATGATTACGAATTCCGGCGTCGGCGGATACC	To generate pK18-Δ*PA1251*
Δ*PA1251*-up-R	CCTCGTCCATCCGTTGTTCCTG	
Δ*PA1251*-down-F	CAGGAACAACGGATGGACGAGGGCCAACGACCTCAAGGGCAT	
Δ*PA1251*-down-R	GCATGCCTGCAGGTCGACTCTAGAGGATCCGGGACGCCCATGCCGATT	
Δ*bdlA*-up-F	GGAAACAGCTATGACCATGATTACGAATTCCCAATCCTGCGGTATTCAACG	To generate pK18-Δ*bdlA*
Δ*bdlA*-up-R	GGCGTACTTGACCACCTTGAGC	
Δ*bdlA*-down-F	GCTCAAGGTGGTCAAGTACGCCCGCCGAGCAGACCAACCT	
Δ*bdlA*-down-R	GCATGCCTGCAGGTCGACTCTAGAGGATCCGGTGCAGAGCCCCTACGAACT	
Δ*aer*-up-F	GGAAACAGCTATGACCATGATTACGAATTCCATGCCGCTCAGCGTGGAA	To generate pK18-Δ*aer*

Δ*aer*-up-R	TCGTAGATCGGCGTGACATAGGC	
Δ*aer*-down-F	GCCTATGTCACGCCGATCTACGACACCATCTGATCGCCAAGTTGC	
Δ*aer*-down-R	GCATGCCTGCAGGTCGACTCTAGAGGATCCCCTGGGGCACAGCCTCTATCATAT	
Δ*PA1608*-up-F	GGAAACAGCTATGACCATGATTACGAATTCAACACCCACGGCACCTCCAC	
Δ*PA1608*-up-R	AGGAAGCGTTTGCTGCTGTCG	To generate
Δ*PA1608*-down-F	CGACAGCAGCAAACGCTTCCTCCGCCAGCCAGGAACTGTC	pK18-Δ*PA1608*
Δ*PA1608*-down-R	GCATGCCTGCAGGTCGACTCTAGAGGATCCGCGTGCGGTAGGGCGAATAA	
Δ*PA1646*-up-F	GGAAACAGCTATGACCATGATTACGAATTCCGACGAGCGCCATGTGGAT	
Δ*PA1646*-up-R	AGCAGGACCAGGGTAAAACCGA	To generate
Δ*PA1646*-down-F	TCGGTTTTACCCTGGTCCTGCTCGCGAGGTGGCCGATCAG	pK18-Δ*PA1646*
Δ*PA1646*-down-R	GCATGCCTGCAGGTCGACTCTAGAGGATCCGGGCCTGATCGACGTGCAG	
Δ*mcpS*-up-F	GGAAACAGCTATGACCATGATTACGAATTCAAGCCACCGATGCTGCCGTT	
Δ*mcpS*-up-R	CCGATGGTGCTTGCCGACC	To generate
Δ*mcpS*-down-F	GGTCGGCAAGCACCATCGGGTGGTCACCGAGAACGTCAAGC	pK18-Δ*mcpS*
Δ*mcpS*-down-R	GCATGCCTGCAGGTCGACTCTAGAGGATCCGGCAAGGCGTCTTCCACAGC	
Δ*ctpH*-up-F	GGAAACAGCTATGACCATGATTACGAATTCAGGCGACCTACAACCGTCTGC	
Δ*ctpH*-up-R	CATGGAACGCCTCGATGGATT	To generate
Δ*ctpH*-down-F	AATCCATCGAGGCGTTCCATGTCCCGCAACCTCACGGAAAT	pK18-Δ*ctpH*
Δ*ctpH*-down-R	GCATGCCTGCAGGTCGACTCTAGAGGATCCTGGTCGTGAGCCTGTCCCTGAT	
Δ*PA2573*-up-F	GGAAACAGCTATGACCATGATTACGAATTCGTTTCGGCCAGTCGGTCTACCA	
Δ*PA2573*-up-R	ATGTCGCCTTCCATGTGGTTGC	To generate
Δ*PA2573*-down-F	GCAACCACATGGAAGGCGACATAGCGTTCGCAGCAGAACACCC	pK18-Δ*PA2573*
Δ*PA2573*-down-R	GCATGCCTGCAGGTCGACTCTAGAGGATCCTCGCCCTCGGACCTGCTCTATC	
Δ*PA2562*-up-F	GGAAACAGCTATGACCATGATTACGAATTCTTTCGTGGTCTTCTCGCTGTCC	
Δ*PA2562*-up-R	GCTCGCGGGTATCCTTGACTT	To generate
Δ*PA2562*-down-F	AAGTCAAGGATACCCGCGAGCGGAGATCAACCGCAGCGTGC	pK18-Δ*PA2562*
Δ*PA2562*-down-R	GCATGCCTGCAGGTCGACTCTAGAGGATCCTGATGCCGAAGCTGATGACGAA	
Δ*tlpQ*-up-F	GGAAACAGCTATGACCATGATTACGAATTCGGATACCGCCACGCAGAGC	To generate
Δ*tlpQ*-up-R	GGAAGGCATCCATGAAATAGCG	pK18-Δ*tlpQ*
Δ*tlpQ*-down-F	CGCTATTTCATGGATGCCTTCCCATCGCCGAGCAGACCAAC	

Δ*tlpQ*-down-R	GCATGCCTGCAGGTCGACTCTAGAGGATCCGGTGTTCCTGTTGGGCGAGT	
Δ*PA2788*-up-F	GGAAACAGCTATGACCATGATTACGAATTCGGTTGTCCTGCTCGCTCTGCA	To generate pK18-Δ*PA2788*
Δ*PA2788*-up-R	GCTCAGGTACAACGCCACCGA	
Δ*PA2788*-down-F	TCGGTGGCGTTGTACCTGAGCGTCGCCACGGTGGAAGAACTG	
Δ*PA2788*-down-R	GCATGCCTGCAGGTCGACTCTAGAGGATCCCACCACGCTGAACTGGACCCT	
Δ*PA2867*-up-F	GGAAACAGCTATGACCATGATTACGAATTCCGACCTGCACATCGGCATCAC	To generate pK18-Δ*PA2867*
Δ*PA2867*-up-R	CGGATCAGCAGTTGCGAGGC	
Δ*PA2867*-down-F	GCCTCGCAACTGCTGATCCGCGCAGATGCGTGAAAGCAACAC	
Δ*PA2867*-down-R	GCATGCCTGCAGGTCGACTCTAGAGGATCCAGGCAGCCCCAGGCAACAG	
Δ*PA2920*-up-F	GGAAACAGCTATGACCATGATTACGAATTCTTTCGCCATCGGCCCG	To generate pK18-Δ*PA2920*
Δ*PA2920*-up-R	GTTTTCCGCGTGATCGTTCCT	
Δ*PA2920*-down-F	AGGAACGATCACGCGGAAAACCGACGAAATCGCCCGCA	
Δ*PA2920*-down-R	GCATGCCTGCAGGTCGACTCTAGAGGATCCCACCCGTGCCGCTTCCA	
Δ*PA4290*-up-F	GGAAACAGCTATGACCATGATTACGAATTCGGCGGCATAAAGCGTCACTTC	To generate pK18-Δ*PA4290*
Δ*PA4290*-up-R	CTGGCTGGTGTTATGGGACAGG	
Δ*PA4290*-down-F	CCTGTCCCATAACACCAGCCAGAGCAAGCGCCTGTTCAACGAC	
Δ*PA4290*-down-R	GCATGCCTGCAGGTCGACTCTAGAGGATCCCTCACCCTGTTCGCCGTGCT	
Δ*pctC*-up-F	GGAAACAGCTATGACCATGATTACGAATTCGCGTCTCGCTGTCCGTGTCA	To generate pK18-Δ*pctC*
Δ*pctC*-up-R	CGGTCAGGGTGCCGATTTCC	
Δ*pctC*-down-F	GGAAATCGGCACCCTGACCGGGCGAGATCGACAGCATGAAC	
Δ*pctC*-down-R	GCATGCCTGCAGGTCGACTCTAGAGGATCCCACCGCCAAGGAGCAGAAATC	
Δ*pctA*-up-F	GGAAACAGCTATGACCATGATTACGAATTCGCAGCGGGCCCATCTCGCTGAT	To generate pK18-Δ*pctA*
Δ*pctA*-up-R	TCCGGGCTGTGGTCGCGGG	
Δ*pctA*-down-F	CCCGCGACCACAGCCCGGAGATCGACGGGATGAACCAGTC	
Δ*pctA*-down-R	GCATGCCTGCAGGTCGACTCTAGAGGATCCCCGAATGGCTGGCCGCCCTG	
Δ*pctB*-up-F	GGAAACAGCTATGACCATGATTACGAATTCATCCAACTGGGGCTGTTCACGA	To generate pK18-Δ*pctB*
Δ*pctB*-up-R	TGTCCTTGTCCACCGAGATGCC	
Δ*pctB*-down-F	GGCATCTCGGTGGACAAGGACATGGACATCACCGAGATCAACACC	
Δ*pctB*-down-R	GCATGCCTGCAGGTCGACTCTAGAGGATCCGGCAGGTCGGAGT	

	GGTAGAAGC	
Δ*PA4520*-up-F	GGAAACAGCTATGACCATGATTACGAATTCGCTTCTTCGCGGTGATCTTCTG	To generate pK18-Δ*PA4520*
Δ*PA4520*-up-R	TCTGCAACAGATCGTTGAGGGA	
Δ*PA4520*-down-F	TCCCTCAACGATCTGTTGCAGATGCAGCAGGCACTGGAGAATAT	
Δ*PA4520*-down-R	GCATGCCTGCAGGTCGACTCTAGAGGATCCGCAGTCGGTCAGTTGGTCGTAG	
Δ*PA4633*-up-F	GGAAACAGCTATGACCATGATTACGAATTCGGACCGCAGTTGGCGTTGA	To generate pK18-Δ*PA4633*
Δ*PA4633*-up-R	CGTCAGCGGATACTCCAACTCG	
Δ*PA4633*-down-F	CGAGTTGGAGTATCCGCTGACGCCACCGAAGAAATCCAGAGCATG	
Δ*PA4633*-down-R	GCATGCCTGCAGGTCGACTCTAGAGGATCCATGTTCCGCGAGGCGTTCC	
Δ*ctpL*-up-F	GGAAACAGCTATGACCATGATTACGAATTCAGCCTGACCGGCCTGTACAACC	To generate pK18-Δ*ctpL*
Δ*ctpL*-up-R	CGGCGGTAGCTCCGGCAA	
Δ*ctpL*-down-F	TTGCCGGAGCTACCGCCGCGATCCATACCATCGGCGTGAT	
Δ*ctpL*-down-R	GCATGCCTGCAGGTCGACTCTAGAGGATCCCCGGCGAGGAAGAAGAACAGC	
Δ*PA4915*-up-F	GGAAACAGCTATGACCATGATTACGAATTCGCGAACAACTGGTGGCGGT	To generate pK18-Δ*PA4915*
Δ*PA4915*-up-R	TTGAGCTGGCTGATGGGCTTC	
Δ*PA4915*-down-F	GAAGCCCATCAGCCAGCTCAACCACGGCGACGGAAGAACA	
Δ*PA4915*-down-R	GCATGCCTGCAGGTCGACTCTAGAGGATCCGCTGGTCGCTGAGCGTGTTCTA	
Δ*mcpK*-up-F	GGAAACAGCTATGACCATGATTACGAATTCCTTCTGGCAGACCCCGTTCCT	To generate pK18-Δ*mcpK*
Δ*mcpK*-up-R	GTTCCATCAGGCTGGCGACTT	
Δ*mcpK*-down-F	AAGTCGCCAGCCTGATGGAACACAGCATCACCCGCACCGT	
Δ*mcpK*-down-R	GCATGCCTGCAGGTCGACTCTAGAGGATCCGGCGCAGTTCTTCGACAATCC	
C*pctA*-F	GATAACAATTTCACACAGGAAACAGAATTC ATGATCAAAAGTCTGAAGTTCAGCCA	To generate pME6032-*pctA*
C*pctA*-R	CTGATCCGCTAGTCCGAGGCCTCGAGATCTTCAGATCTTGAAGCTGTCCACCA	
C*tlpQ*-F	GATAACAATTTCACACAGGAAACAGAATTC ATGTTCCTTCGCCGCCTGT	To generate pME6032-*tlpQ*
C*tlpQ*-R	CTGATCCGCTAGTCCGAGGCCTCGAGATCTTCAGGCCTTGAACTGTTCCATC	
C*pctB*-F	GATAACAATTTCACACAGGAAACAGAATTC ATGATCAAAAGTCTCAAGTTCAGCC	To generate pME6032-*pctB*
C*pctB*-R	CTCACTGATCCGCTAGTCCGAGGCCTCGAG TCAGATCTTGAAGCTGTCCACCA	

pctA-LBD-F<br>*pctA-LBD*-R	ACTGGTGGACAGCAAATGGGTCGCGGATCCAACGATTACCTGCAGCGCAA<br>GTGGTGGTGCTCGAGTGCGGCCGCAAGCTTTCAGGCCGAGACGCGGAACT	To generate pET28a-*pctA-LBD*
pctB-LBD-F<br>*pctB-LBD*-R	ACTGGTGGACAGCAAATGGGTCGCGGATCCAACGACTCCCTGCAGCGTG<br>GTGGTGGTGCTCGAGTGCGGCCGCAAGCTTTCACGAGGTACGCAGCTTGGTGA	To generate pET28a-*pctB-LBD*
pctC-LBD-F<br>*pctC-LBD*-R	ACTGGTGGACAGCAAATGGGTCGCGGATCCAACGATTACCGACAGCGCG<br>GTGGTGGTGCTCGAGTGCGGCCGCAAGCTTTCACGCCGAAGTGCGGAATT	To generate pET28a-*pctC-LBD*
tlpQ-LBD-F<br>*tlpQ-LBD*-R	GGAAGATCTATGCTCGACGAATCGGCGCGCCTG<br>CCCAAGCTTTCACAGGGCCGGGCCGAGCAGG	To generate pET28a-*tlpQ-LBD*
mcpX-LBD-F<br>*mcpX-LBD*-R	ACTGGTGGACAGCAAATGGGTCGCGGATCCCTCATCTCCCAGACGCAG<br>GTGGTGGTGCTCGAGTGCGGCCGCAAGCTTTCAGCCTTCGCCGTCGTAT	To generate pET28a-*mcpX-LBD*
soHK1S-Z6-LBD-F<br>*soHK1S-Z6-LBD*-R	ACTGGTGGACAGCAAATGGGTCGCGGATCCATTGAGAAACGGCTTTATGAGAATCT<br>GTGGTGGTGCTCGAGTGCGGCCGCAAGCTTTCACACAACTACACGCCATTTTAGCTTC	To generate pET28a-*soHK1S-Z6-LBD*
vpHK1S-Z8-LBD-F<br>*vpHK1S-Z8-LBD*-R	ACTGGTGGACAGCAAATGGGTCGCGGATCCGAAAACACCGCCAAAGAAG<br>GTGGTGGTGCTCGAGTGCGGCCGCAAGCTTTCAGATGAGATGCAAAGGCTCG	To generate pET28a-*vpHK1S-Z8-LBD*
kinD-LBD-F<br>*kinD-LBD*-R	ACTGGTGGACAGCAAATGGGTCGCGGATCCATAGCTGCAGAACATAAACAAGAAG<br>GTGGTGGTGCTCGAGTGCGGCCGCAAGCTTTCAGCTGACAGGTTTCTGATCCTCT	To generate pET28a-*kinD-LBD*
rpHK1S-Z16-LBD-F<br>*rpHK1S-Z16-LBD*-R	ACTGGTGGACAGCAAATGGGTCGCGGATCCAAGGGCTACGACTCGCACAAG<br>GTGGTGGTGCTCGAGTGCGGCCGCAAGCTTTCAGTCGGAACGCACCGCCTC	To generate pET28a-*rpHK1S-Z16-LBD*
lsrB-F<br>*lsrB*-R	ACTGGTGGACAGCAAATGGGTCGCGGATCCGCAGAGCGTATTGCATTTATTC<br>GTGGTGGTGCTCGAGTGCGGCCGCAAGCTTTCAGAAATCGTATTTGCCGA	To generate pET28a-*lsrB*
cheR1-F<br>*cheR1*-R	ACTGGTGGACAGCAAATGGGTCGCGGATCCGTGTCGGCAGCTAATGCGG<br>GTGCTCGAGTGCGGCCGCAAGCTTGTCGACCTACTTGGCCCGGTAGATGATG	To generate pET28a-*cheR1*
pctA-F<br>*pctA-his*$_6$-R	GATCGGATCCATGATCAAAAGTCTGAAGTTCAGC<br>GATCAAGCTTTCAATGGTGATGGTGATGATGGATCTTGAAGCTGTCCAC	To generate pHSe5-*pctA-his*$_6$

kinD-F	CGCGGATCCATGTTGGAGCGATGCAAAT	To generate pHSe5-*kinD-his*$_6$
kinD-his$_6$-R	CCCAAGCTTCTAATGGTGATGGTGATGATGTGATGCGGATACGGGG	
rpHK1S-Z16-F	CGCGGATCCATGCCCCCTCGCCCTCTC	To generate pHSe5-*rpHK1S-Z16-his*$_6$
rpHK1S-Z16-his$_6$-R	CCCAAGCTTCTAATGGTGATGGTGATGATGCGAGGTGGCGTCGGCT	
Y101A-up-F	GCTTGCGACATCCATTCATCCT	To generate *pctA-LBD*^{Y101A}
Y101A-up-R	GGTGAAGACGCCGTCCTGCTGGCCGAGGGCGGTGAAG	
Y101A-down-F	CTTCACCGCCCTCGGCCAGCAGGACGGCGTCTTCACC	
Y101A-down-R	TGTTGGTGCGGTTGGACTGC	
M111A-up-F	AAGGATTCGGCGAGGTGCTG	To generate *pctA-LBD*^{M111A}
M111A-up-R	GCCGGCATCGGGCTGTCCGGACGCGCGGTGAAGA	
M111A-down-F	TCTTCACCGCGCGTCCGGACAGCCCGATGCCGGC	
M111A-down-R	TGTTGGTGCGGTTGGACTGCT	
Y121A-up-F	CGCTTGCGACATCCATTCATC	To generate *pctA-LBD*^{Y121A}
Y121A-up-R	GTCCTTGTACCAGGGCCGGCTGCGCGGATCGGCG	
Y121A-down-F	CGCCGATCCGCGCAGCCGGCCCTGGTACAAGGAC	
Y121A-down-R	GACGCTGTTGGTGCGGTTG	
R126A-up-F	ACATCCATTCATCCTCGGGACTAA	To generate *pctA-LBD*^{R126A}
R126A-up-R	CCGGCCGCCACGGCGTCCTTGTACCAGGGCGCG	
R126A-down-F	CGCGCCCTGGTACAAGGACGCCGTGGCGGCCGG	
R126A-down-R	GACGCTGTTGGTGCGGTTG	
W128A-up-F	GCTTGCGACATCCATTCATCCT	To generate *pctA-LBD*^{W128A}
W128A-up-R	CCGCCGGCCGCCACGGCGTCCTTGTACGCG	
W128A-down-F	CGCGTACAAGGACGCCGTGGCGGCCGGCGG	
W128A-down-R	ATGTTCACCGTGCGGCTGT	
Y144A-up-F	GCTTGCGACATCCATTCATCCT	To generate *pctA-LBD*^{Y144A}
Y144A-up-R	CCTGGGTGGCGGCGTCGACGGCGGGTTCGGTCA	
Y144A-down-F	TGACCGAACCCGCCGTCGACGCCGCCACCCAGG	
Y144A-down-R	GCTGTTGGTGCGGTTGGACT	
D146A-up-F	GAAGGATTCGGCGAGGTGCT	To generate *pctA-LBD*^{D146A}
D146A-up-R	CGGTGATGATCAATTCCTGGGTGGCGGCGGCGACGTAG	
D146A-down-F	TACGTCGCCGCCGCCACCCAGGAATTGATCATCACCG	
D146A-down-R	GCTGTTGGTGCGGTTGGACT	
A147F-up-F	CCATTCATCCTCGGGACTAAAGC	To generate *pctA-*LBD^{A147F}
A147F-up-R	CGGTGATGATCAATTCCTGGGTGGCGAAGTCGACGTAGGGTTCG	
A147F-down-F	CGAACCCTACGTCGACTTCGCCACCCAGGAATTGATCATCACCG	
A147F-down-R	TGTTGGTGCGGTTGGACTGCT	
D173A-up-F	GCTTGCGACATCCATTCATCCT	To generate *pctA-LBD*^{D173A}
D173A-up-R	ATTGATGATCTGCACCAGGGTCTTCAGGCTGAGGGCGCCG	
D173A-down-F	CGGCGCCCTCAGCCTGAAGACCCTGGTGCAGATCATCAAT	
D173A-down-R	GACGCTGTTGGTGCGGTTG	

E146D-up-F	AACGACTCCCTGCAGCGTG	To generate *pctB*-LBD^{E146D}
E146D-up-R	CAGCTCGTGGATGGCCGGATCCATGTAGGGTTCGGTGAG	
E146D-down-F	CTCACCGAACCCTACATGGATCCGGCCATCCACGAGCTG	
E146D-down-R	CGAGGTACGCAGCTTGGTGA	
F147Y-up-F	AACGATTACCGACAGCGCGA	To generate *pctC-LBD*^{F147Y}
F147Y-up-R	CGAGGATCTGCTCGCCGGTACCGGCATCGACGTAAGGTTCG	
F147Y-down-F	CGAACCTTACGTCGATGCCGGTACCGGCGAGCAGATCCTCG	
F147Y-down-R	CGAAGTGCGGAATTCGCTGAG	
W192A-up-F	GCCTGTCCATCCAATGGAAGAT	To generate *tlpQ-LBD*^{W192A}
W192A-up-R	GCAGGGTTGCCCGCTTTCCTTCGGGCAGGTGTACGC	
W192A-down-F	GCGTACACCTGCCCGAAGGAAAGCGGGCAACCCTGC	
W192A-down-R	CGGCAGACGCTGGGTCAG	
Y208A-up-F	GCCGCCTGTCCATCCAAT	To generate *tlpQ-LBD*^{Y208A}
Y208A-up-R	AGCAGTTGGCGTTCGCCGACCTTGTCGAAGGCAGGATC	
Y208A-down-F	GATCCTGCCTTCGACAAGGTCGGCGAACGCCAACTGCT	
Y208A-down-R	GTCGCGCAGCATGTGGG	
D210A-up-F	CCAATGGAAGATCACCCTGCT	To generate *tlpQ*-LBD^{D210A}
D210A-up-R	ATGCTGGTCATCAGCAGTTGGCGTTCGCCGACCTTGGC	
D210A-down-F	GCCAAGGTCGGCGAACGCCAACTGCTGATGACCAGCAT	
D210A-down-R	GGCAGACGCTGGGTCAGG	
D239A-up-F	GGAAGATCACCCTGCTGGCA	To generate *tlpQ-LBD*^{D239A}
D239A-up-R	GGTTGCTCAGGTTGATGGCCTTCGCTGAGCGCCTGCA	
D239A-down-F	GGCCATCAACCTGAGCAACCTGCAGGCGCTCAGCGAA	
D239A-down-R	CGGCAGACGCTGGGTCAG	
pfs-F	GAAGTTCTGTTCCAGGGGCCCCTGGGATCCATGAAAATCGGCATCATTG	To generate pGEX-6P-1-*pfs*
pfs-R	GTCAGTCACGATGCGGCCGCTCGAGTCGACTTAGCCATGTGCCAGTTTCT	
luxS-F	GAAGTTCTGTTCCAGGGGCCCCTGGGATCCATGCCGTTGTTAGATAGCTTC	To generate pGEX-6P-1-*luxS*
luxS-R	GTCAGTCACGATGCGGCCGCTCGAGTCGACCTAGATGTGCAGTTCCTGCA	
Δ*luxS*-sg20-F	CTCCTAGGTATAATACTAGTGGTTTTTATATGAGTCTGATGTTTTAGAGCTAGAAATAGC	To generate pTargetF1-Δ*luxS*
Δ*luxS*-sg20-R	CTCAAAAAAAGCACCGACTCGG	
Δ*luxS*-up-F	CCGAGTCGGTGCTTTTTTTGAGATCTGACTTTCTCTGCCCGTA	
Δ*luxS*-up-R	TGAAGCTATCTAACAACGGCA	
Δ*luxS*-down-F	TGCCGTTGTTAGATAGCTTCACTGCCGAAAGAGAAGTTGC	
Δ*luxS*-down-R	ACGCGTCGACACGCTGGCGGGGTCT	
Q87IU5-LBD-F	ACTGGTGGACAGCAAATGGGTCGCGGATCCTCTCAAACTCAGGAGCACATCAATT	To generate pET28a-*Q87IU5-LBD*
Q87IU5-LBD-R	GTGCTCGAGTGCGGCCGCAAGCTTGTCGACTCAGATTGCGCCCA	

	CGTACCAG	
Q8E8U9-LBD-F	ACTGGTGGACAGCAAATGGGTCGCGGATCCTCATTGTTGCTGCAAAATAGTTTAG	To generate pET28a-*Q8E8U9-LBD*
Q8E8U9-LBD-R	GTGCTCGAGTGCGGCCGCAAGCTTGTCGACTCACACTATCACCAGTTCCCAATCG	
Q92RJ4-LBD-F	ACTGGTGGACAGCAAATGGGTCGCGGATCCGCCACGACCACGAAAGCAG	To generate pET28a-*Q92RJ4-LBD*
Q92RJ4-LBD-R	GTGCTCGAGTGCGGCCGCAAGCTTGTCGACTCACGCGAAGCCGACATACCAT	

***Underlined sites indicate restriction enzyme cutting sites added for cloning.**

Supplementary References

1. Rico-Jiménez, M. *et al.* Paralogous chemoreceptors mediate chemotaxis towards protein amino acids and the non-protein amino acid gamma-aminobutyrate (GABA). *Mol. Microbiol.* **88**, 1230–1243 (2013).
2. Gavira, J. A. *et al.* How bacterial chemoreceptors evolve novel ligand specificities. *mBio* **11**, e03066-19 (2020).
3. Corral-Lugo, A. *et al.* High-affinity chemotaxis to histamine mediated by the TlpQ chemoreceptor of the human pathogen *Pseudomonas aeruginosa. mBio* **9**, e01894-18 (2018).
4. Zhang, Y. & Skolnick, J. TM-align: a protein structure alignment algorithm based on the TM-score. *Nucleic Acids Res.* **33**, 2302–2309 (2005).
5. Bibikov, S. I., Miller, A. C., Gosink, K. K. & Parkinson, J. S. Methylation-independent aerotaxis mediated by the *Escherichia coli* Aer protein. *J. Bacteriol.* **186**, 3730–3737 (2004).
6. Lu, M. *et al.* Transcriptome response to heavy metals in *Sinorhizobium meliloti* CCNWSX0020 reveals new metal resistance determinants that also promote bioremediation by Medicago lupulina in metal-contaminated soil. *Appl Environ Microbiol.* **83**, e01244-17 (2017).
7. Turick, C. E. *et al.* The role of 4-hydroxyphenylpyruvate dioxygenase in enhancement of solid-phase electron transfer by *Shewanella oneidensis* MR-1. *FEMS Microbiol. Ecol.* **68**, 223–225 (2009).
8. Yang, N., Liu, M., Luo, X. & Pan, J. Draft genome sequence of Strain ATCC 17802^T, the type strain of *Vibrio parahaemolyticus. Mar. Genomics* **24**, 203–205 (2015).

9. Simon, R., Priefer, U. B. & Pühler, A. A broad host range mobilization system for in vivo genetic engineering: transposon mutagenesis in gram negative bacteria. *Nat. Biotechnol.* **1**, 784–791 (1983).

10. Xu, L. *et al.* A cyclic di-GMP-binding adaptor protein interacts with a chemotaxis methyltransferase to control flagellar motor switching. *Sci. Signal.* **9**, ra102 (2016).

11. Miller, S. T. *et al. Salmonella* typhimurium recognizes a chemically distinct form of the bacterial quorum-sensing signal AI-2. *Mol. Cell.* **15**, 677–687 (2004).

12. Dennis, J. J. & Zylstra, G. J. Plasposons: modular self-cloning minitransposon derivatives for rapid genetic analysis of gram-negative bacterial genomes. *Appl. Environ. Microbiol.* **64,** 2710-2715 (1998).

13. Schafer, A., *et al.* Small mobilizable multi-purpose cloning vectors derived from the *Escherichia coli* plasmids pK18 and pK19: selection of defined deletions in the chromosome of *Corynebacterium glutamicum. Gene* **145**, 69–73 (1994).

14. Heeb, S., Blumer, C. & Haas, D. Regulatory RNA as mediator in GacA/RsmA-dependent global control of exoproduct formation in *Pseudomonas fluorescens* CHA0. *J. Bacteriol.* **184**, 1046–1056 (2002).

15. Jiang, Y. *et al.* Multigene editing in the *Escherichia coli* genome via the CRISPR-Cas9 system. *Appl. Environ. Microbiol.* **81**, 2506–2514 (2015).

16. Zhang, L. *et al.* The catabolite repressor/activator Cra is a bridge connecting carbon metabolism and host colonization in the plant drought resistance-promoting bacterium *Pantoea alhagi* LTYR-11Z. *Appl. Environ. Microbiol.* **84**, e00054-18 (2018).
