# Supplementary material for: Sensing of autoinducer-2 by functionally distinct receptors in prokaryotes
Source: Nat Commun. 2020 Oct 23;11:5371. doi: 10.1038/s41467-020-19243-5 (PMC7584622; doi:10.1038/s41467-020-19243-5)
Supplement: Supplementary file 3 — Description of Additional Supplementary Files [file 41467_2020_19243_MOESM3_ESM.pdf]

Description of Additional Supplementary Files

File Name: Supplementary Data 1
Description: **Domain predictions of dCache_1-containing proteins by PfamScan against the Pfam database.** 18970 dCache_1-containing proteins were downloaded from Pfam 32.0 database based on Uniprot 2018_04 release, and domain predictions with PfamScan were carried out at domain E-value threshold of 1E-5. Uniprot accessions, protein length, Pfam domain architectures, output domains, the organisms from which the dCache_1-containing proteins were taken and their taxonomy are listed. For each dCache_1 domain, the starting and ending position in protein, domain length and domain E-value are given.

File Name: Supplementary Data 2
Description: **Domain predictions of dCache_1-containing proteins with all the five conserved residues corresponding to R126, W128, Y144, D146 and D173 of PctA.** Multiple alignment analysis of 18970 dCache_1 domains was performed with ClustalW embedded in MEGA7, and 1535 dCache_1 domains with all the five conserved residues corresponding to R126, W128, Y144, D146 and D173 of PctA were found. The domain prediction results for proteins containing this type of dCache_1 domains were extracted from Supplementary Data 1 and presented.

File Name: Supplementary Data 3
Description: **Multiple alignment on the sequences of 18970 dCache_1 domains.** The amino acid sequences of 18970 dCache_1 domains were downloaded from Pfam 32.0 database as a text file in FASTA format. The FASTA sequence file was subjected to the MEGA7 software and multiple alignment analysis was performed with ClustalW embedded in MEGA7.
