# Supplementary material for: Sensing of autoinducer-2 by functionally distinct receptors in prokaryotes
Source: Nat Commun. 2020 Oct 23;11:5371. doi: 10.1038/s41467-020-19243-5 (PMC7584622; doi:10.1038/s41467-020-19243-5)
Supplement: Supplementary file 7 — Reporting Summary [file 41467_2020_19243_MOESM7_ESM.pdf]

nature research

Corresponding author(s): Lei Zhang, Zhao-Qing Luo and Xihui Shen

Last updated by author(s): Sep 16, 2020

Reporting Summary

Nature Research wishes to improve the reproducibility of the work that we publish. This form provides structure for consistency and transparency in reporting. For further information on Nature Research policies, see our Editorial Policies and the Editorial Policy Checklist.

Statistics

For all statistical analyses, confirm that the following items are present in the figure legend, table legend, main text, or Methods section.

n/a	Confirmed	
☐	☒	The exact sample size (*n*) for each experimental group/condition, given as a discrete number and unit of measurement
☐	☒	A statement on whether measurements were taken from distinct samples or whether the same sample was measured repeatedly
☐	☒	The statistical test(s) used AND whether they are one- or two-sided<br>*Only common tests should be described solely by name; describe more complex techniques in the Methods section.*
☒	☐	A description of all covariates tested
☒	☐	A description of any assumptions or corrections, such as tests of normality and adjustment for multiple comparisons
☐	☒	A full description of the statistical parameters including central tendency (e.g. means) or other basic estimates (e.g. regression coefficient) AND variation (e.g. standard deviation) or associated estimates of uncertainty (e.g. confidence intervals)
☐	☒	For null hypothesis testing, the test statistic (e.g. *F*, *t*, *r*) with confidence intervals, effect sizes, degrees of freedom and *P* value noted<br>*Give P values as exact values whenever suitable.*
☒	☐	For Bayesian analysis, information on the choice of priors and Markov chain Monte Carlo settings
☒	☐	For hierarchical and complex designs, identification of the appropriate level for tests and full reporting of outcomes
☒	☐	Estimates of effect sizes (e.g. Cohen's *d*, Pearson's *r*), indicating how they were calculated

Our web collection on statistics for biologists contains articles on many of the points above.

Software and code

Policy information about availability of computer code

Data collection	Nano ITC Standard Volume isothermal calorimeter (TA Instruments, New Castle, DE), Revolution XD laser-scanning confocal microscope (Andor, Belfast, Northern Ireland), microplate reader Victor X3 (PerkinElmer, Waltham, MA, USA), AB SCIEX Triple Quad 6500+ LC-MS/MS System, Optima ultracentrifuge XPN-100 and rotor 70 Ti (Beckman Coulter, USA), EASY-nLC 1000 and Orbitrap Fusion Tribrid mass spectrometer (Thermo Fisher Scientific), HPLC (Agilent 1260 infinity II) equipped with a C18 reversed-phase column and a UV detector
Data analysis	Prism Graphpad 7.0, NanoAnalyze v3.4, Chimera v1.13, Schrödinger suite (version 2018-4) and the Protein Preparation Wizard tool implemented in the Schrödinger suite, Glide v8.1, LigPrep v2.5, Mascot Daemon v2.5.1, MEGA7, ClustalW v2.1, Imaris 9.0, COMSTAT v2.1, ImageJ v1.48, TM-align (version 20190822), HHpred (https://toolkit.tuebingen.mpg.de/tools/hhpred), PfamScan v1.6, Weblogo 3, TMHMM v2.0

For manuscripts utilizing custom algorithms or software that are central to the research but not yet described in published literature, software must be made available to editors and reviewers. We strongly encourage code deposition in a community repository (e.g. GitHub). See the Nature Research guidelines for submitting code & software for further information.

Data

Policy information about availability of data

All manuscripts must include a data availability statement. This statement should provide the following information, where applicable:

- Accession codes, unique identifiers, or web links for publicly available datasets
- A list of figures that have associated raw data
- A description of any restrictions on data availability

The protein sequence and domain data are available from the Pfam database (http://pfam.xfam.org/) and Uniprot database (http://www.uniprot.org/). Protein 3D coordinate data are available from the Protein Data Bank (http://www.rcsb.org). All the other data that support the findings of this study are available within the

paper and its Supplementary Information and Supplementary Data or from the corresponding authors upon reasonable request. The source data underlying Figs. 1b–e, 2, 3, 4a, b, d and 5 and Supplementary Figs. 3, 5, 7, 8b and 11 are provided as a Source Data file.

Field-specific reporting

Please select the one below that is the best fit for your research. If you are not sure, read the appropriate sections before making your selection.

- [x] Life sciences
- [ ] Behavioural & social sciences
- [ ] Ecological, evolutionary & environmental sciences

For a reference copy of the document with all sections, see nature.com/documents/nr-reporting-summary-flat.pdf

Life sciences study design

All studies must disclose on these points even when the disclosure is negative.

Sample size	Sample sizes were determined without statistical measures, but based on widely used sizes in relevant publications within this field of research (Chew et al. mBio 2018, 9:e00585-18; Corral-Lugo et al. mBio 2018, 9:e01894-18; Gan et al. Nat Microbiol. 2019, 4:134-143; Lin et al. Nat. Commun. 2017, 8:14888; Torcato et al. J. Biol. Chem. 2019, 294:4450-4463) to ensure that it will be appropriate for statistical analysis.
Data exclusions	No data were excluded from the analyses.
Replication	All experimental findings reported in the paper were reliably performed for at least 3 times, and all the attempts at replication were successful.
Randomization	Randomization is not applicable in this study as it employed bacterial strains.
Blinding	Blinding was not relevant in this study as it employed bacterial strains.

Reporting for specific materials, systems and methods

We require information from authors about some types of materials, experimental systems and methods used in many studies. Here, indicate whether each material, system or method listed is relevant to your study. If you are not sure if a list item applies to your research, read the appropriate section before selecting a response.

Materials & experimental systems

n/a	Involved in the study	
x		Antibodies
x		Eukaryotic cell lines
x		Palaeontology and archaeology
x		Animals and other organisms
x		Human research participants
x		Clinical data
x		Dual use research of concern

Methods

n/a	Involved in the study	
x		ChIP-seq
x		Flow cytometry
x		MRI-based neuroimaging
